# Chain-Branched Polyhydroxylated Octahydro-*1H*-Indoles as Potential Leads against Lysosomal Storage Diseases

**DOI:** 10.3390/ph12020047

**Published:** 2019-03-29

**Authors:** Juan C. Estévez, Marcos A. González, M. Carmen Villaverde, Yuki Hirokami, Atsushi Kato, Fredy Sussman, David Reza, Ramón J. Estévez

**Affiliations:** 1 Centro Singular de Investigación en Química Biolóxica e Materiais Moleculares, Universidade de Santiago de Compostela, 15782 Santiago de Compostela, Spain; juancarlos.estevez@usc.es (J.C.E.); mar.gonzalez.castro@gmail.com (M.A.G.); fredy.sussman@usc.es (F.S.); david.reza@gmail.com (D.R.); 2 Departamento de Química Orgánica, Universidade de Santiago de Compostela, 15782 Santiago de Compostela, Spain; mc.villaverde@usc.es; 3 Department of Hospital Pharmacy, University of Toyama, Toyama 930-0194, Japan; yuhi@saitama-med.ac.jp (Y.H.); kato@med.u-toyama.ac.jp (A.K.)

**Keywords:** sugars, iminosugars, glycosidase inhibition

## Abstract

Here, the synthesis and glycosidase inhibition properties of the two first known 3-ethyloctahydro-1*H*-indole-4,5,6-triols are reported. This study shows the transformation of d-glucose into polyhydroxylated 1-(2-nitrocyclohexane) acetaldehydes, followed by a protocol involving the formation of the azacyclopentane ring. Results of inhibitory potency assays and docking calculations show that at least one of them could be a lead for optimization in the search for compounds that behave like folding chaperones in lysosomal storage diseases.

## 1. Introduction

**Figure 1:**
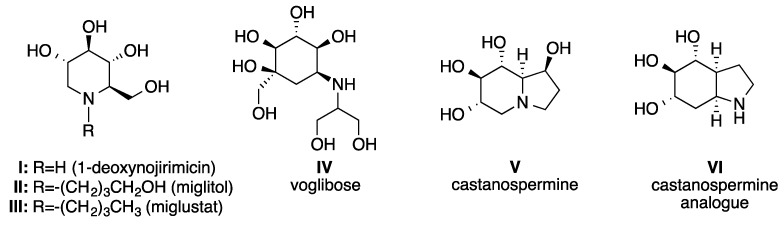
Selection of iminosugars and aminocarbasugars.

Iminosugars [1,2,3,4] and aminocarbasugars [5,6,7] are sugar mimics that inhibit a variety of enzymes of therapeutic interest, including glycosidases and glycosyltransferases. They have been shown to be lead molecules for the treatment of diseases such as diabetes, viral infections, or lysosomal storage disorders. Some representative examples are included in Figure 1. Thus, the *N*-alkylated 1-deoxynojirimycin miglitol (**II**) (Glyset^®^) [8,9] and the aminocarbasugar voglibose (**IV**) [10,11] have been approved for the treatment of type II diabetes [12,13], and miglustat (**III**) (Zavesca^®^) is prescribed for the treatment of Gaucher disease [14,15]. The bicyclic iminosugar castanospermine (**V**) [16,17] is a polyhydroxylated indolizidine alkaloid [3,18] that can be considered as a conformational restricted analogue of miglitol. This compound and its derivatives have received considerable attention as potential antineoplastic and immunosuppressive agents, but, unfortunately, they have shown toxicity to human cells [19,20,21,22]. This led to the study of analogues of these leads aimed at altering their activity and toxicity profile. Specifically, the polyhydroxylated octahydroindole (**VI**) and its *N*-hydroxyalkyl derivatives were studied as castanospermine analogues where the position of the *N*-atom was changed, allowing them to be considered as conformational restricted carbasugars [23,24].

As a contribution to the search for new castanospermine analogues, and as a part of ongoing project on synthetic application of nitro sugars and related compounds [25], we report herein the synthesis and biological evaluation of compounds **10** and **13** as novel polyhydroxylated octahydroindoles bearing an alkyl substituent at the C-3 position. Results of the inhibitory potency assays and docking calculations have shown that at least one of them could be a lead for optimization in the search for compounds that behave like folding chaperones, useful as drugs against lysosomal storage diseases like G_M1_ gangliosidosis and Morquio B disease [26].

## 2. Results

### 2.1. Chemical Synthesis

Michael addition of carbonyl synthetic equivalents to electron-deficient nitro alkenes has proven to be a promising method for the enantioselective synthesis of γ−nitro acids [27,28]. In this regard, carbasugar nitro olefins are suitable scaffolds for this and other synthetic purposes [29], although they are practically unexplored. They include in their structure a preformed carbocyclic ring bearing several hydroxy substituents in a well-defined spatial orientation and a nitroethylene subunit suitable for Michael addition of nucleophiles.

In order to apply this methodology, the key six-membered nitroolefin **4b** was obtained from the known nitro sugar **1** [30], according to a protocol developed by us for the synthesis of the first polyhydroxylated 1-nitrocyclohexane [31].

**Scheme 1:**
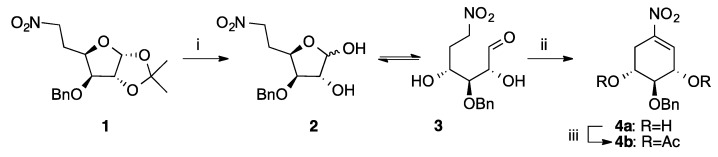
Conditions: (**i**) TFA/H_2_O, rt, 5 h. (**ii**) 2% K_2_CO_3_/MeOH, rt, 4 h. (**iii**) Ac_2_O, DMAP (cat), Et_2_O, rt, 6 h (77%, three steps).

Removal of the isopropylidene protecting group of **1** under acidic conditions [trifluoracetic acid (TFA)/H_2_O] was followed by a potassium carbonate-promoted intramolecular Henry reaction of the resulting ε-nitro aldehyde **3** (the open form of the tricomponent mixture **2** + **3**), which was directly reacted with potassium carbonate in methanol followed by acetic anhydride and a catalytic amount of dimethoxyaminopyridine (DMAP) in order to promote its dehydration aimed at obtaining the cyclic nitroolefin **4b**, via **4a** (Scheme 1).

**Scheme 2:**
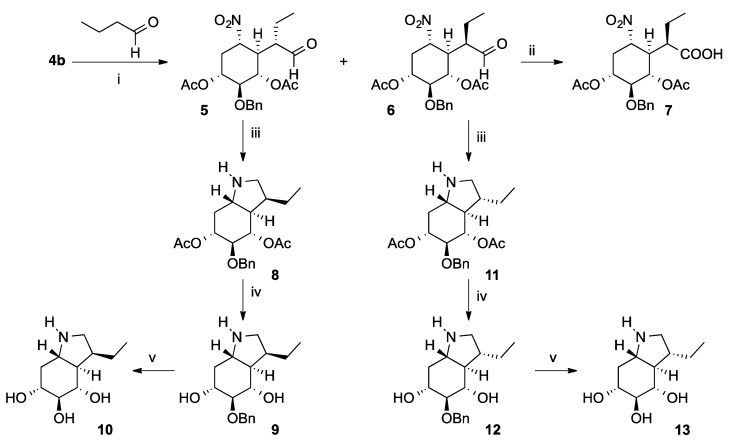
Conditions: (**i**) pyrrolidine, CH_2_Cl_2_, rt, 3 h (50% for **5**, 21% for **6**); (**ii**) NaClO_2_/NaH_2_PO_4_, 2-methyl-2-butene, *t*-BuOH/H_2_O (3:1), rt, 2 h, 88%; (**iii**) Zn dust, MeOH/AcOH (1:1), 0 °C, 14 h (90% for **8**, 85% for **11**); (**iv**) 1 M NaOH/MeOH, rt, 4 h /69% for **9** and for **12**); (**v**) 10% Pd/C, MeOH, H_2_ (1 atm) (87% for **10**, 88% for **13**).

Michael addition of butyraldehyde to **4b**, catalyzed by pyrrolidine, resulted in an epimeric mixture of γ-nitro aldehydes **5** and **6**, which were isolated by column chromatography (Scheme 2). The stereochemical outcome of this reaction, which is controlled by the substrate, can be rationalized on the basis of the Felkin–Ahn rule.

**Figure 2:**
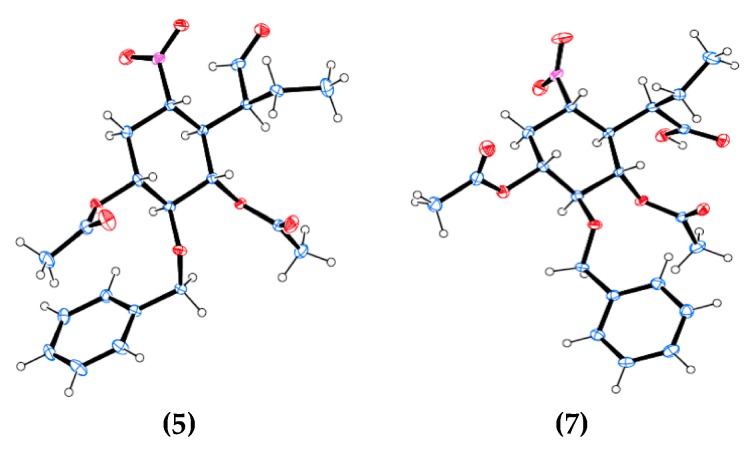
X-ray structures of compounds **5** and **7**.

The structure of compounds **5** and **6** were unambiguously identified by X-ray experiments (Figure 2). In the latter case this was corroborated by the X-ray structure of the corresponding carboxylic acid **7**, which was obtained by (2,2,6,6-tetramethylpiperidin-1-yl)oxyl (TEMPO) oxidation of **6**.

Finally, γ−nitroaldehydes **5** and **6** were satisfactorily converted into the corresponding octahydroindoles **10** and **13** (Scheme 2). Reaction of compound **5** with Zn dust in an acidic media for 14 h directly provided the corresponding octahydroindole **8**, by the reduction of the nitro group to amino, followed by a spontaneous reductive amination of the resulting γ−amino aldehyde. Basic hydrolysis of **8** with NaOH resulted in a selective hydrolysis of its acetate subunits to give derivative **9**. Catalytic hydrogenation of **9** gave the trihydroxylated octahydroindole **10**. On the other hand, the epimeric octahydroindole **13** was similarly obtained from γ−nitro aldehyde **6** via compounds **11** and **12**.

### 2.2. Glycosidase Inhibition Assays

Table 1 displays results of the inhibition of a variety of glycosidases by iminosugars **10** and **13**, as the inhibition percentage at 1000 μM. As seen from this table, these compounds display by far the highest inhibition activity against bovine β-galactosidase (amongst all enzymes assayed), albeit at levels under 50%.

### 2.3. Docking Studies

To further shed some light on the differences between the inhibition activities of these compounds for lysosomal β-galactosidase (β-Gal), we performed docking simulations that included the above mentioned compounds **10** and **13**, as well as galactose, a catalytic product of this enzyme. We included this latter compound as a point of reference, since the structure of its complex with human β-galactosidase (with whom the bovine variant shares a highly conserved binding domain) is known.

The docking results for these compounds are summarized in Table 2. This table lists the scoring value for the top-scoring poses obtained in three docking calculations that differ in the options used in this work, which include “early termination search”, “diverse solutions”, and docking runs that allow for active site side chain flexibility (see methods section for details). The table also includes the hydrogen bond pattern between the ligands and some residues of the protein.

**Figure 3:**
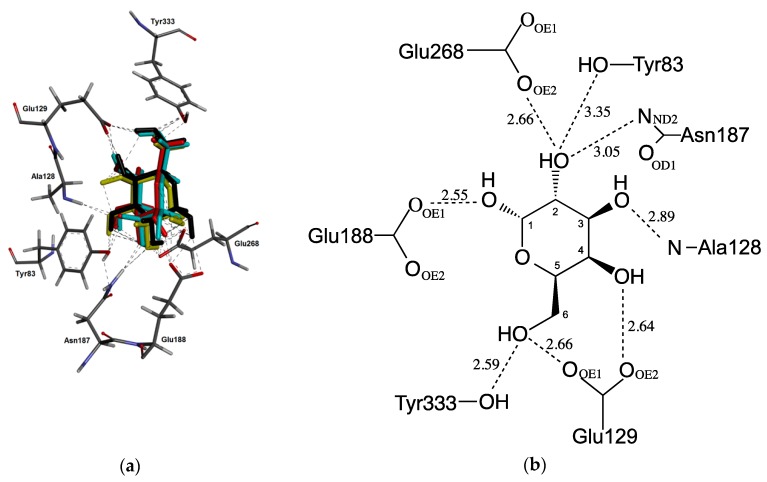
(**a**) Top galactose poses obtained in early termination runs with the various scoring functions used, superimposed on the one observed in the crystal structure, drawn in yellow. The ChemScore structure is drawn in red, while the GoldScore and ChemPLP are drawn in black and cyan, respectively. (**b**) Hydrogen bond interactions between the ligand and the protein residues, resulting from the GoldScore scoring protocol run, observed as well in PDB entry 3THC [32].

**Figure 4:**
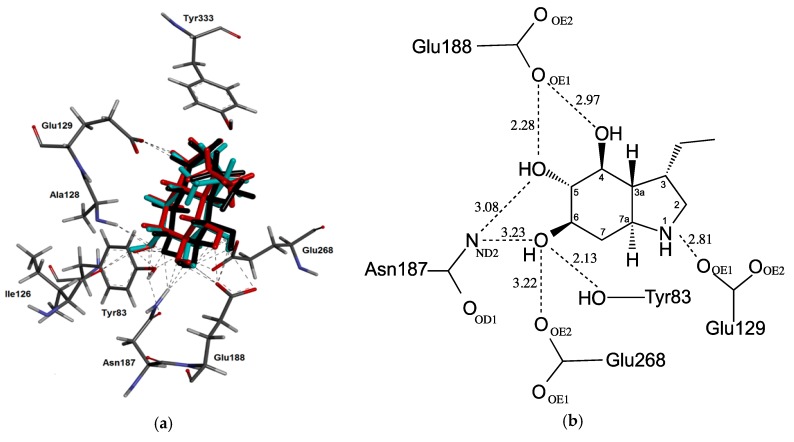
(**a**) Top poses obtained for compound **10** in early termination runs with the various scoring functions used. The ChemScore structure is drawn in red, while the GoldScore and ChemPLP are drawn in black and cyan, respectively. (**b**) Hydrogen bond interactions between the ligand and the protein residues, resulting from the GoldScore scoring protocol run.

**Figure 5:**
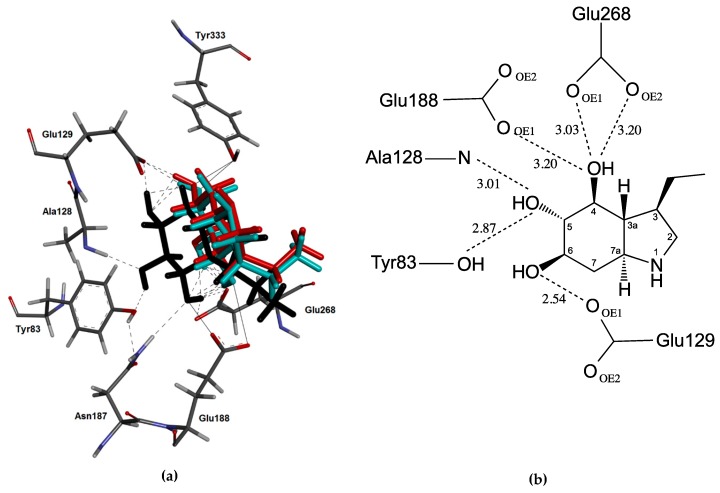
(**a**) Top poses obtained for compound **13** in early termination runs with the various scoring functions used. The ChemScore structure is drawn in red, while the GoldScore and ChemPLP are drawn in black and cyan, respectively. (**b**) Hydrogen bond interactions between the ligand and the protein residues, resulting from the GoldScore scoring protocol run.

In this table we have used a color code for every scoring value as well as the hydrogen bond pattern. It is used to identify common poses obtained across different docking simulations. Compounds whose poses share the same color in different docking runs have a similar top exit pose. For instance, most of the scoring values in the docking results for galactose are colored with a single color (green), to indicate that there seems to be a consensus on the pose reached by these compounds, which reproduces the one found in the crystal structure of the β-galactosidase-galactose complex found in Protein Data Bank (PDB) entry 3HTC (see Figure 3). This outcome serves to validate the docking protocol used for these calculations. Docking of ligand **10** produces a similar pose in most of the cases (see Figure 4), colored cyan in Table 2. Compound **13** displays a larger number of different poses than compound **10**, indicating this latter ligand fits better in the binding site, a result that may underlie its higher inhibitory activity (see Table 1). Figure 5 shows the poses that result for compound **13** using all the three scoring functions. Most of the poses obtained for this ligand are un-colored, in order to point out that these are single binding conformations which do not display comparable poses amongst the different docking runs. The only pose that is reproduced in more than one run (for this compound) was obtained with GoldScore and has the largest number of hydrogen bonds.

To further asses the uniqueness of the binding poses for the three ligands used in our studies we have reviewed the “early termination” protocol results. In this protocol the best pose search is terminated as soon as a specified number of runs have a similar solution, within a given Root Mean Square Deviation (RMSD) of each other (see methods section). The results show that the number of runs increases for the weaker binder (compound **13**) as compared to compound **10**, indicating a lack of consensus for the best pose for the former. For instance, compound **13** does not find a consensus solution with the ChemPLP scoring function in the early termination run. Finally, cluster analysis results indicate that the latter compound present fewer clusters at the RMSD cut-off used in this study (results not shown), an outcome that further validates the hypothesis behind the difference in affinity.

## 3. Discussion

This work presents new synthetic routes to polyhydroxylated octahydroindoles with pharmacological chaperone (PC) therapeutic potential aimed at autosomal recessive diseases like G_M1_ gangliosidosis and Morquio B, a group of lysosomal storage disorders resulting from the abnormal metabolism of macro-substances such as glycosphingolipids, glycogen, mucopolysaccharides, and glycoproteins [33]. This abnormal metabolism may result in neurodegenerative disorders as well as dwarfism and skeletal abnormalities [34].

These diseases are caused by mutations in the lysosomal β-galactosidase (β-Gal), which are frequently related to misfolding and subsequent endoplasmic reticulum-associated degradation [26]. PC therapy is a novel approach that uses small molecule ligands of the mutant enzyme to promote the correct folding and prevent endoplasmic reticulum-associated degradation while promoting trafficking to the lysosome [33]. The affinity of the leads for the enzyme should not be high since it is desirable that the activity of the enzyme should be preserved. The range of affinities for these compounds usually span from the milli- to the micro-molar level. Some of the efficacious leads are galactose analogues. In this family of ligands the hydroxyl groups serve to maintain the structural binding specificity. The compounds studied here (**10** and **13**) are polyhydroxylated octahydroindoles and hence represent a departure from the galactose analogue paradigm. The results indicate that these compounds display inhibitory potency only for β-Gal at a milli-molar level, making them a good starting point for further development as PCs. The docking calculations performed herein for these compounds have allowed us to rationalize the small difference in binding between these compounds. As seen above, compound **10** displays a single well defined binding pose, while its epimer **13** loses this pose specificity, an outcome that may explain the observed binding affinity order.

The β-galactosidase mutations that originate the four phenotypes of the G_M1_ gangliosidosis and Morquio B diseases have been identified and have been mapped into the crystallographic structure of this molecule. Most mutations fall outside the active site and hence will not disturb the ligand–protein interactions. Actually, one of the phenotypes of this disease does not present any mutations in the active site. The other three phenotypes each present a single mutation that could affect the hydrogen bond pattern described above or ligand–protein van der Waals contacts [34]. As a conclusion, the present work opens new venues for PC therapies other than galactose analogs. It will be desirable in the future to carry out the binding kinetics and docking studies of our compounds with the few known β-galactosidase active site mutants in order to advance in the lead compound generation.

## 4. Materials and Methods

### 4.1. Methods for the Glycosidase Inhibition Studies

The enzymes α-glucosidase (from yeast, rice), β-glucosidase (from almonds, bovine liver), α-galactosidase (from coffee beans), β-galactosidase (from bovine liver), α-mannosidase (from jack beans), β-mannosidase (from snails), α-l-fucosidase (from bovine kidney), α-l-rhamnosidase (from *Penicillium decumbens*), β-glucuronidases (from *Escherichia coli*), α,α-trehalase (from porcine kidney), amyloglucosidase (from *Aspergillus niger*), *p*-nitrophenyl glycosides, and various disaccharides were purchased from Sigma-Aldrich Co. Brush border membranes were prepared from the rat small intestine according to the method of Kessler et al. [35], and were assayed at pH 6.8 for rat intestinal maltase using maltose. For rat intestinal glucosidases and porcine kidney trehalase activities, the reaction mixture (0.2 mL) contained 25 mM substrate and the appropriate amount of enzyme, and the incubations were performed for 10 min at 37 °C. The reaction was stopped by heating at 100 °C for 3 min. After centrifugation (600 g; 10 min), 0.035 mL of the resulting reaction mixture were added to 2.1 mL of the Glucose CII-test Wako (Wako Pure Chemical Ind., Osaka, Japan). The absorbance at 505 nm was measured to determine the amount of the released d-glucose. Other glycosidase activities were determined using an appropriate *p*-nitrophenyl glycoside as substrate at the optimum pH of each enzyme. The reaction mixture (0.2 mL) contained 2 mM of the substrate and the appropriate amount of enzyme. The reaction was stopped by adding 0.4 mL of 400 mM Na_2_CO_3_. The released *p*-nitrophenol was measured spectrometrically at 400 nm.

### 4.2. Docking Protocol

We carried out the docking simulations with the suite of modules resident in the program GOLD [36]. We scored the poses with three of the scoring resident functions (i.e., GoldScore [37,38], ChemScore [39,40,41], and ChemPLP [42]).

For the docking predictions to our target we used the X-ray structure of human β-galactosidase (hβ-Gal) bound to galactose (PDB entry 3THC) [34]. As a first step we selected the catalytic domain (residues 29 to 360) and we cleaned up the target structure discarding alternative conformations and adding hydrogen atoms, using the Discovery Studio (DS) modules [43]. We defined the binding site as including all the β-Gal catalytic domain atom residues that lay at 6 Å from the ligand (i.e., β-galactose). We performed the docking simulations using three different approaches in the GOLD “Fitness Search Options” running setup: in the first one we used the “allow early termination” option, which instructs GOLD to terminate docking of a ligand as soon as a specified number of runs have a similar solution, within a given RMSD of each other. In the second one we used the “generate diverse solutions” option in which diversity is reinforced during the ligand mapping stage. This aim is reached by comparing the RMSD of the current solution against those that have already been generated. If the RMSD is below the diversity threshold or the maximum of solutions per cluster has been reached, the mapping is rejected and the process repeated until an acceptable solution is generated. In the last one we used the “allow early termination” option but leaving flexibility of the side chains of the active site residues Tyr83, Glu129, Asn187, Glu188, Glu268, and Tyr333. In all cases the docking conformations generated by the genetic algorithm were evaluated by the all three scoring functions mentioned above. In the case of galactose, we validated our methodology by searching for poses similar to the crystallographic one. In the case of ligands **10** and **13** we performed an additional analysis, searching (across all docking options) for hydrogen bond interactions similar to the ones observed in the galactose complex.

### 4.3. Chemical Synthesis Methods

Melting points were determined using a Kofler Thermogerate apparatus and are uncorrected. Specific rotations were recorded on a JASCO DIP-370 optical polarimeter, infrared spectra on a MIDAC FTIR spectrophotometer, and nuclear magnetic resonance spectra on a Bruker WM-250 or a Varian Mercury 300 apparatus. Mass spectra were obtained on a Kratos MS 50 TC mass spectrometer. Elemental analyses were obtained from the Elemental Analysis Service at the University of Santiago de Compostela. Thin layer chromatography (tlc) was performed using Merck GF-254 type 60 silica gel and ethyl acetate/hexane mixtures as eluents; the tlc spots were visualized with Hanessian mixture. Column chromatography was carried out using Merck type 9385 silica gel. Solvents were purified as in reference [44].

#### 4.3.1. (1R,2S,3S)-2-(Benzyloxy)-5-nitrocyclohex-4-ene-1,3-diyl diacetate (**4b**)

A 2:1 TFA/H_2_O mixture (90 mL) was added to compound **1** (285 mg, 0.88 mmol) and the resulting mixture was stirred at room temperature for 5 h. The solvents were evaporated under vacuum and co-evaporated with toluene (3 × 10 mL). The crude was solved in methanol (20 mL), 2% aq. K_2_CO_3_ (9 mL) were added, and the mixture was stirred at room temperature for 4 h. The reaction was then neutralized with DOWEX 50WX4-50 (previously acidified at pH = 1), filtered and concentrated to dryness, to provide **4a**.

Ac_2_O (1.8 mL, 19.4 mmol) and a catalytic amount of DMAP (30 mg) were added to a solution of crude **4a** in Et_2_O (60 mL) and the resulting solution was stirred at room temperature for 12 h. The reaction was then concentrated to dryness and the residue was solved in CH_2_Cl_2_ (30 mL) and washed with H_2_O (3 × 15 mL). The organic layer was dried (anhydrous Na_2_SO_4_), filtered and concentrated to dryness. The residue was purified by flash column chromatography (AcOEt/Hex 1:3) to give compound **4b** (237 mg, 77% yield), as an amorphous white solid. [α]_D_^20^: +69.5 (c 2.2, CHCl_3_). ^1^H-RMN (Cl_3_CD, 250 MHz, ppm): 1.96 (s, 3H, CH_3_); 1.98 (s, 3H, CH_3_); 2.66–2.77 (m, 1H, H-6); 2.99–3.10 (m, 1H, H-6′); 3.81 (dd, *J* = 6.9 Hz, *J* = 4.3 Hz, 1H); 4.64 (s, 2H, CH_2_Ph); 5.15 (dt, *J* = 7.0, *J* = 5.3 Hz, 1H, H-1); 5.51 (tt, *J* = 4.1 Hz, *J* = 1.9 Hz, 1H, H-3); 6.99 (d, *J* = 3.4 Hz, 1H, H-4); 7.17–7.32 (m, 5H, 5xH-Ar). ^13^C-RMN (Cl_3_CD, 62.5 MHz, ppm): 20.8 (CH_3_); 20.9 (CH_3_); 27.4 (CH_2_); 67.9 (CH); 68.7 (CH); 73.7 (CH_2_); 75.1 (CH); 127.7 (CH); 127.8 (2xCHAr); 128.2 (CHAr); 128.6 (2xCHAr); 137.4 (C); 148.2 (C); 169.7 (CO); 170.0 (CO). IR (ν, cm^−1^): 1747 (s, C=O); 1528 (m, NO_2_), 1340 (m, NO_2_). HRMS (ESI^+^): calculated for C_17_H_19_NNaO_7_ [M + Na]^+^: 372.1054. Found: 372.1061.

#### 4.3.2. (1R,2S,3S,4R,5S)-2-(Benzyloxy)-5-nitro-4-((S)-1-oxobutan-2-yl)cyclohexane-1,3-diyl diacetate (**5**) and (1R,2S,3S,4R,5S)-2-(benzyloxy)-5-nitro-4-((R)-1-oxobutan-2-yl)cyclohexane-1,3-diyl diacetate (**6**)

n-Butanal (211 µL, 2.34 mmol) and pyrrolidine (99 µL, 1.21 mmol) were added to a solution of nitroolefin **4b** (157 mg, 0.45 mmol) in CH_2_Cl_2_ (4 mL). The resulting solution was stirred at room temperature for 3 h and then was concentrated to dryness. Flash column chromatography of the residue (EtOAc/hexane 1:4) provided compound **5** (98 mg, 50% yield, white solid) and its epimer **6** (21% yield, clear gum).

Compound **5**. m.p.: 112.5–114.0 °C (EtOAc/Hex). [α]_D_^20^ = −17.3° (c 1.5, CHCl_3_). ^1^H–RMN (Cl_3_CD, 250 MHz, ppm): 0.88 (t, *J* = 7.3 Hz, 3H, CH_2_CH_3_); 1.13–1.33 (m, 2H, CH_2_CH_3_); 1.74 (s, 3H, CH_3_); 1.92 (s, 3H, CH_3_); 1.75–2.13 (m, 2H, H-6+H-6′); 2.63 (dt, *J* = 4.2 Hz; *J* = 12.2 Hz, 1H, H-4); 2.92 (td, *J* = 1.9 Hz; *J* = 11.5 Hz, 1H, H-2a); 3.54 (t, *J* = 9.4 Hz, 1H, H-2); 4.54 (ABq, *J* = 11.4 Hz, 2H, CH_2_Ph); 4.61–4.93 (m, 3H, H-1 + H-3 + H-5); 7.12–7.28 (m, 5H, 5xAr-H); 9.44 (d, *J* = 1.1 Hz, 1H, H-C=O). ^13^C-RMN (Cl_3_CD, 62.5 MHz, ppm): 13.5 (CH_3_); 16.0 (CH_2_); 20.6 (CH3); 20.9 (CH_3_); 33.8 (CH_2_); 44.0 (CH); 52.3 (CH); 69.1 (CH); 70.3 (CH); 75.1 (CH_2_); 81.5 (CH); 82.2 (CH); 127.7 (2xCH-Ar); 128.0 (CH-Ar); 128.6 (2xCH-Ar); 137.6 (C); 169.1 (CO); 169.8 (CO); 200.1 (CO). IR (ν, cm^−1^): 1745 (s, C=O); 1556 (s, NO_2_), 1369 (s, NO_2_). MS-ESI^+^ (m/z, %): 444.1 (100, [M + Na]^+^). EA: Calculated for C_21_H_27_NO_8_: C, 59.85; H, 6.46; N, 3.32. Found: C, 59.84; H, 6.54; N, 3.16.

Compound **6**. [α]_D_^20^ = −1.9° (c 1.0, CHCl_3_). ^1^H-RMN (Cl_3_CD, 250 MHz, ppm): 0.89 (t, *J* = 7.1 Hz, 3H, -CH_2_CH_3_); 1.15–1.52 (m, 2H, -CH_2_CH_3_); 1.90 (s, 3H, CH_3_); 1.91 (s, 3H, CH_3_); 1.74–2.07 (m, 2H, H-6 + H-6′); 2.51 (dt, *J* = 12.1 Hz, *J* = 4.41 Hz,1H, H-4); 2.75–2.85 (m, 1H, H-2a); 3.58 (t, *J* = 9.2 Hz, 1H, H-2); 4.52-4.67 (m, 3H, CH_2_Ph + H-5); 4.81 (ddd, *J* = 4.6 Hz; *J* = 9.5 Hz; *J* = 11.6 Hz, 1H, H-1); 5.01 (dd, *J*_3,2_ = 9.2 Hz; *J*_3,4_ = 11.1 Hz, 1H, H-3); 7.14–7.30 (m, 5H, 5xAr-H); 9.44 (s, 1H, H-C=O). ^13^C-RMN (Cl_3_CD, 62.5 MHz, ppm): 13.2 (CH_3_); 17.4 (CH_2_); 20.9 (CH_3_); 21.0 (CH_3_); 33.9 (CH_2_); 43.5 (CH); 51.5 (CH); 70.2 (CH); 70.5 (CH); 75.2 (CH_2_); 80.6 (CH); 81.9 (CH); 127.6 (2xCH-Ar); 128.0 (CH-Ar); 128.6 (2xCH-Ar); 137.8 (C); 169.8 (2xCO); 201.3 (CO). IR (ν, cm^−1^): 1752 (s, C=O); 1718 (s, C=O), 1555 (s, NO_2_), 1372 (s, NO_2_). HRMS (ESI^+^): Calculated for C_21_H_27_NNaO_8_ [M + Na]^+^: 444.1629. Found: 444.1623.

#### 4.3.3. (R)-2-((1R,2S,3S,4R,6S)-2,4-Diacetoxy-3-(benzyloxy)-6-nitrocyclohexyl)butanoic acid (**7**)

2-Methyl-2-butene (115 µL, 1.09 mmol), NaH_2_PO_4_.2H_2_O (50 mg, 0.32 mmol) and NaClO_2_ (13.5 mg, 0.15 mmol) were added to a 0 °C cooled solution of aldehyde **6** (115 mg, 0.27 mmol) in a 5:2 ^t^BuOH/H_2_O mixture (7 mL). The reaction was then stirred at room temperature for 2 h, the ^t^BuOH was removed under vacuum, and the resulting mixture was extracted with EtOAc (3 × 10 mL). The organic layers were dried (anhydrous Na_2_SO_4_), filtered, and the solvents were removed under vacuum. The residue was submitted to flash column chomathography (EtOAc/Hexane 1:2) to give compound **7** (105 mg, 88% yield), as a solid mp: 146.8-148.1 °C (EtOAc/Hexane). [α]_D_^20^ = +10.8° (c 1.4, CHCl_3_). ^1^H-RMN (Cl_3_CD, 250 MHz, ppm): 0.92 (t, *J* = 7.2 Hz, 3H, CH_2_CH_3_); 1.38–2.10 (m, 2H, CH_2_CH_3_); 1.94 (s, 3H, CH_3_); 1.98 (s, 3H, CH_3_); 2.18–2.24 (m, 2H, H-5 + H-5′); 2.59 (dt, *J* = 4.6 Hz; *J* = 12.3 Hz, 1H, H-1); 2.80 (td, *J* = 1.9 Hz; *J* = 11.2 Hz, 1H, H-2a); 3.61 (t, *J* = 9.1 Hz, 1H, H-3); 4.57–4.70 (m, 3H, CH_2_Ph + H-6); 4.92 (tdd, *J* = 11.5 Hz, *J* = 8.8 Hz, *J* = 5.1 Hz, 1H, H-2); 5.15 (dd, *J* = 9.0 Hz; *J* = 11.2 Hz, 1H, H-4); 7.20–7.36 (m, 5H, 5xAr-H); 9.24 (sa, 1H CO_2_H). ^13^C-RMN (Cl_3_CD, 62.5 MHz, ppm): 13.0 (CH_3_); 21.0 (2xCH_3_); 21.5 (CH_2_); 34.3 (CH_2_); 45.2 (CH); 45.6 (CH); 70.3 (CH); 70.6 (CH); 74.9 (CH_2_); 82.0 (CH); 82.2 (CH); 127.8 (2xCH-Ar); 127.9 (CH-Ar); 128.6 (2xCH-Ar); 137.9 (C); 169.8 (CO); 169.9 (CO); 177.5 (CO). IR (ν, cm^−1^): 1746 (s, C=O); 1711 (s, C=O); 1557 (s, NO_2_); 1370 (s, NO_2_). HRMS (ESI^+^): Calculated for C_21_H_27_NNaO_9_ [M + Na]^+^: 460.1578. Found: 460.1555.

#### 4.3.4. (3S,3aR,4S,5S,6R,7aS)-5-(Benzyloxy)-3-ethyloctahydro-1H-indole-4,6-diyl diacetate (**8**)

Zinc dust (969 mg, 14.82 mmol) was added to a 0 °C cooled solution of aldehyde **5** (250 mg, 0.59 mmol) in a 1:1 MeOH/AcOH mixture (15 mL) and the resulting suspension was stirred at 0 °C for 14 h. The reaction was filtered through a celite pad, which was eluted with methanol, and the filtrate was concentrated to dryness. The residue was disolved in Cl_2_CH_2_ (15 mL) and was washed with saturated aq. NaHCO_3_ (2x10 mL). The organic layer was dried (anhydrous Na_2_SO_4_), filtered, and concentrated to dryness under vacuum. Flash column chromathography of the resulting oil (CH_2_Cl_2_/MeOH 9:1) provided compound **8** (206 mg, 90% yield,), as a white amorphous solid. [α]_D_^20^ = −10.8 (c 1.8, CHCl_3_). ^1^H-RMN (Cl_3_CD, 250 MHz, ppm): 0.77 (t, *J* = 7.4 Hz, 3H, CH_2_CH_3_); 1.09–1.49 (m, 5H, H-3 + H-7 + H-7′ + CH_2_-CH_3_); 1.90 (s, 3H, CH_3_); 1.91 (s, 3H, CH_3_); 2.06 (bs, 1H, NH); 2.27 (ddd, *J* = 3.4 Hz, *J* = 5.1 Hz, *J* = 11.7 Hz, 1H, H-3a); 2.65–2.74 (m, 2H, H-2 + H-2′); 3.21 (t, *J* = 10.0 Hz, 1H, H-7a); 3.53 (t, *J* = 9.3 Hz, 1H, H-5); 4.58 (ABq, *J* = 11.6 Hz, 2H, CH_2_Ph); 4.89 (ddd, *J* = 5.1 Hz, *J* = 9.5 Hz, *J* = 11.3 Hz, 1H, H-6); 4.99 (dd, *J* = 9.1 Hz, *J* = 11.1 Hz, 1H, H-4); 7.16–7.28 (m, 5H, 5x-Ar-H). ^13^C-RMN (Cl_3_CD, 62.5 MHz, ppm): 12.0 (CH_3_); 21.0 (2xCH_3_); 21.9 (CH_2_); 35.3 (CH_2_); 39.7 (CH); 51.4 (CH_2_); 52.0 (CH); 54.5 (CH); 71.1 (CH); 73.5 (CH); 74.7 (CH_2_); 84.8 (CH); 127.4 (2xCH-Ar); 127.5 (CH-Ar); 128.3 (2xCH-Ar); 138.3 (C); 169.8 (CO); 169.9 (CO). IR (ν, cm^−1^): 3205 (b, NH), 1740 (s, C=O). HRMS (ESI^+^): Calculated for C_21_H_30_NO_5_ [M + H]^+^: 376.2118. Found: 376.2122.

#### 4.3.5. (3S,3aR,4S,5S,6R,7aS)-5-(Benzyloxy)-3-ethyloctahydro-1H-indole-4,6-diol (**9**)

A 1 M NaOH methanolic solution (1 mL, 1 mmol) was added to a solution of amine **8** (30 mg, 0.08 mmol) in methanol (2 mL) and the resulting solution was stirred at room temperature for 4 h. Half of the methanol was then evaporated, and the reaction was neutralized with saturated NH_4_Cl solution and extracted with ethyl acetate (3 × 10 mL). The joined organic layers were dried (anhydrous Na_2_SO_4_), filtered, and concentrated to dryness under vacuum. Purification of the resulting oil by flash column chromathography (CH_2_Cl_2_/MeOH 7:1) gave compound **9** (16 mg, 69% yield), as a clear gum. [α]_D_^20^ = +3.0 (c 1.0, CH_3_OH) ^1^H-RMN (CD_3_OD, 250 MHz, ppm): 0.88 (t, *J* = 7.3 Hz, 3H, CH_2_-CH_3_); 1.19–1.58 (m, 4H); 1.92–2.07 (m, 2H); 2.18 (ddd. *J* = 3.4 Hz, *J* = 5.0 Hz, *J* = 11.7 Hz, 1H, H-3a); 2.68–2.88 (m, 2H, H-2′ + H-7a); 3.15 (t, *J* = 8.8 Hz, 1H); 3.31–3.46 (m, 1H); 3.54–3.64 (m, 1H, H-6); 4.83 (s, 2H, CH_2_Ph); 7.18–7.31 (m, 3H, 3xAr-H); 7.37–7.42 (m, 2H, 2xAr-H). ^13^C-RMN (CD_3_OD, 62.5 MHz, ppm): 12.7 (CH_3_); 28.1 (CH_2_); 36.7 (CH_2_); 45.3 (CH); 52.3 (CH_2_); 54.8 (CH); 59.4 (CH); 72.7 (CH); 75.8 (CH); 76.6 (CH); 90.4 (CH); 128.5 (CH-Ar); 129.2 (4xCH-Ar); 140.5 (C). IR (ν, cm^−1^): 3371 (b, NH + OH). HRMS (ESI^+^): Calculated for C_17_H_26_NO_3_, [M + H]^+^: 292.1907. Found: 292.1902

#### 4.3.6. (3S,3aR,4S,5S,6R,7aS)-3-Ethyloctahydro-1H-indole-4,5,6-triol (**10**)

Pd/C 10% (15 mg) was added to a deoxygenated solution of amine **9** (15 mg, 0.05 mmol) in methanol (2 mL) and the resulting suspension was stirred at room temperature for 6 h, under a hydrogen atmosphere (P = 1 atm). The reaction was then filtered through a celite pad, eluted with methanol, and the filtrate concentrated to dryness. Flash column chromathography of the residue (CH_2_Cl_2_/MeOH 5:1) allowed isolation of compound **10** (9 mg, 87% yield) as a yellow oil. [α]_D_^20^ = +16.4 (c 0.9, MeOH). ^1^H-RMN (CD_3_OD, 250 MHz, ppm): 0.91 (t, *J* = 7.4 Hz, 3H, CH_2_CH_3_); 1.22–1.39 (m, 1H); 1.49–1.69 (m, 2H); 1.91–2.20 (m, 2H), 2.28 (dt, 1H, *J* = 11.5 Hz, *J* = 4.2 Hz, 1H); 2.99–3.19 (m, 3H), 3.40–3.64 (m, 3H). ^13^C-RMN (CD_3_OD, 62,5 MHz, ppm): 12.5 (CH_3_); 28.0 (CH_2_); 33.5 (CH_2_); 39.2 (CH); 42.6 (CH); 62.7 (CH_2_); 66.9 (CH); 72.0 (CH); 75.4 (CH); 81.2 (CH). IR (ν, cm^−1^): 3405 (b, NH + OH). HRMS (ESI+): Calculated for C_10_H_20_NO_3_ [M + H]^+^: 202.1438. Found: 202.1438.

#### 4.3.7. (3R,3aR,4S,5S,6R,7aS)-5-(Benzyloxy)-3-ethyloctahydro-1H-indole-4,6-diyl diacetate (**11**)

Starting from aldehyde **6** (100 mg, 0.24 mmol) and following the same procedure as for compound **8**, compound **11** was obtained (76 mg, 85% yield), as a white amorphous solid. [α]_D_^20^ = +9.3 (c 1.8, CHCl_3_). ^1^H-RMN (Cl_3_CD, 250 MHz, ppm): 0.79–0.93 (m, 4H), 0.97–1.12 (m, 1H), 1.18–1.51 (m, 4 H), 1.76–1.83 (m, 1H), 1,93 (s, 3H, CH_3_), 1,96 (s, 3H, CH_3_), 2.35 (ddd, *J* = 11.6 Hz, *J* = 5.0 Hz, *J* = 3.7 Hz, 1H), 2.80 (td, *J* = 11.2 Hz, *J* = 3.3 Hz, 1H), 3.18–3.24 (m, 1H), 3.53–3.59 (m, 1H), 4.62 (ABq, *J* = 11.6 Hz, 2H, CH_2_Ph), 4.93 (ddd, *J* = 11.4 Hz, *J* = 9.6 Hz, *J* = 4.9 Hz, 1H), 5.13 (dd, *J* = 11.6 Hz, *J* = 8.7 Hz, 1H), 7.21–7.33 (m, 5H, 5xAr-H). ^13^C-RMN (Cl_3_CD, 62.5 MHz, ppm): 12.1 (CH_3_); 21.1 (CH_3_); 21.2 (CH_3_); 22.1 (CH_2_); 35.4 (CH_2_); 39.9 (CH); 51.5 (CH_3_); 52.1 (CH); 54.6 (CH); 71.3 (CH); 73.7 (CH); 74.9 (CH_2_); 85.0 (CH); 127.6 (2xCH); 127.7 (2xCH); 128.5 (CH); 138.4 (C); 170.0 (CO); 170.1 (CO). IR (ν, cm^−1^): 3220 (b, NH); 1741 (s, C=O). HRMS (ESI^+^): Calculated for C_21_H_30_NO_5_ [M + H]^+^: 376.2118. Found: 376.2122.

#### 4.3.8. (3R,3aR,4S,5S,6R,7aS)-5-(Benzyloxy)-3-ethyloctahydro-1H-indole-4,6-diol (**12**)

Reaction of amine **11** (54 mg, 0.14 mmol) under the same conditions as for the preparation of amine **9** gave compound **12** (29 mg, 69% yield), as a clear gum. [α]_D_^20^ = +18.2 (c 2.1, CH_3_OH). ^1^H-RMN (CD_3_OD, 250 MHz, ppm): 0.92 (t, *J* = 7.3 Hz, 3H, CH_2_CH_3_); 1.13 (tq, *J* = 13.4 Hz, *J* = 7.0 Hz, 1H, H-CHCH_3_); 1.27–1.48 (m, 1H, H-CHCH_3_); 1.61 (td, *J* = 11.4 Hz, *J* = 7.6 Hz, 1H, H-7); 1.76 (dtd, *J* = 14.9 Hz, *J* = 7.5 Hz, *J* = 3.3 Hz,1H, H-7′); 2.11–2.25 (m, 2H, H-3 + H-3a); 2.61 (td, *J* = 11.6 Hz, *J* = 3.5 Hz, 1H, H-7a + H-2); 2.77 (dd, *J* = 11.5 Hz, *J* = 3.2 Hz, 1H); 3.19–3.27 (m, 2H, H-2 + H-5); 3.52–3.65 (m, 2H); 4.87–4.91 (m, 2H, CH_2_Ph); 7.22–7.37 (m, 3H, 3xAr-H), 7.46–7.49 (m, 2H, 2xAr-H). ^13^C-RMN (CD_3_OD, 62.5 MHz, ppm): 12.4 (CH_3_); 23.1 (CH_2_); 38.7 (CH_2_); 41.1 (CH); 52.1 (CH_2_); 55.2 (CH); 56.0 (CH); 71.8 (CH); 73.1 (CH); 76.5 (CH); 91.1 (CH_2_); 128.5 (2xCH); 129.2 (3xCH); 140.6 (C). IR (ν, cm^−1^): 3371 (b, NH + OH). HRMS (ESI+): Calculated forC_17_H_26_NO_3_ [M + H]^+^: 292.1907. Found: 292.1909.

#### 4.3.9. (3R,3aR,4S,5S,6R,7aS)-3-ethyloctahydro-1H-indole-4,5,6-triol (**13**)

Following the same procedure as for amine **9**, amine **12** (18 mg, 0.06 mmol) gave compound **13** (11 mg, 88% yield), as a yellow solid. [α]_D_^20^ = +36.4 (c 1.1, CH_3_OH). ^1^H-RMN (CD_3_OD, 250 MHz, ppm): 0.90 (t, 3H, *J* = 7.3 Hz, CH_2_CH_3_); 1.10–1.43 (m, 3H); 1.81–1.96 (m, 2H); 2.11–2.46 (m, 4H); 3.13–3.67 (m, 3H). ^13^C-RMN (CD_3_OD, 62.5 MHz, ppm): 12.1 (CH_3_); 24.1 (CH_2_); 35.2 (CH); 38.3 (CH_2_); 52.5 (CH); 63.6 (CH_2_); 63.7 (CH); 71.5 (CH); 72.2 (CH); 82.3 (CH). IR (ν, cm^−1^): 3408 (b, NH + OH). HRMS (ESI^+^): Calculated for C_10_H_20_NO_3_ [M + H]^+^: 202.1438. Found: 202.1438.

X-ray crystal structure for compound **5,** X-ray crystal structure for compound **7**, ^1^H NMR and ^13^C NMR spectra for compounds **4b**, **5**, **6**, **7**, **8**, **9**, **10**, **11**, **12**, and **13** are in Supplementary Materials.

## Figures and Tables

**Table 1:** Data on inhibition of various glycosidases by compounds **10** and **13.**

Enzyme	Compound 10	Compound 13	Miglitol (IC_50_)
α-Glucosidase			
Yeast	16% ^a^	19.5% ^a^	70 μM
Rice	0% ^a^	0% ^a^	0.17 μM
Rat intestinal maltase	4.1% ^a^	0% ^a^	>1000 μM
β-Glucosidase			
Almond	12% ^a^	5% ^a^	>1000 μM
Bovine liver	0% ^a^	21.7% ^a^	>1000 μM
α-Galactosidase			
Coffee beans	15.9% ^a^	0% ^a^	>1000 μM
	0% ^a^	21.7% ^a^	
β-Galactosidase			
Bovine liver	45.6% ^a^	35.8% ^a^	>1000 μM
Lactase	0.6% ^a^	7.4% ^a^	>1000 μM
α-Mannosidase			
Jack bean	0% ^a^	0% ^a^	
β-Mannosidase			
Snail	0% ^a^	2.3% ^a^	>1000 μM
α-L-Fucosidase			
Bovine kidney	7.5% ^a^	12.9% ^a^	>1000 μM
α-L-Rhamnosidase			
*Penicillium decumbens*	0% ^a^	0% ^a^	803
β-Glucronidase			
*E.coli*	18.1% ^a^	12.3% ^a^	>1000 μM
α,α-Trehalase			
Porcine kidney	0% ^a^	0% ^a^	131
Amyloglucosidase			
*Aspergillus niger*	0% ^a^	0% ^a^	>1000 μM

^a^ inhibition % at 1000 μM.

**Table 2:** Results of the docking simulations and score values for the top pose of each run.

Ligand	Protein Residue HB	Fitness & Search Options ^a^									
		Early Termination			Diverse Solutions			Side Flexible Search			
		CHEM	GOLD	PLP	CHEM	GOLD	PLP	CHEM	GOLD	PLP	PDB
**Galactose**	**Galactose score**	**21.88**	**54.49**	**55.63**	**18.57**	**50.17**	**52.84**	**12.57**	**53.60**	**42.29**	**-**
	Tyr83(OH)	3-OH	3-OH	3-OH		3-OH	3-OH	3-OH		3-OH	3-OH
	Ala128(N)		3-OH				3-OH	3-OH	3-OH	3-OH	3-OH
	Glu129(OE1)	6-OH	6-OH	4,6-OH	6-OH	4-OH	4,6-OH	4,6-OH	4-OH	4-OH	6-OH
	Glu129(OE2)	4-OH	4-OH	4-OH	4-OH	4-OH	4-OH		6-OH	6-OH	4-OH
	Asn187(ND2)	2-OH	2-OH	2-OH	2-OH	2-OH	2-OH		2-OH		2-OH
	Asn187(OD)							2-OH			
	Glu188(OE1)	1-OH	1-OH	1-OH	1-OH	1-OH	1-OH		1-OH	1-OH	1-OH
	Glu188(OE2)		1-OH				1-OH	1-OH		1,2-OH	1-OH
	Glu268(OE1)	2-OH	2-OH				2-OH		2-OH	2-OH	2-OH
	Glu268(OE2)	2-OH	2-OH	2-OH	2-OH	2-OH	2-OH		2-OH		2-OH
	Tyr333(OH)	6-OH	6-OH	6-OH	6-OH	6-OH	6-OH		6-OH	6-OH	6-OH
**10**	**10 score**	**27.58**	**44.31**	**49.82**	**27.19**	**44.25**	**45.78**	**20.47**	**52.90**	**38.17**	
	Tyr83(OH)	6-OH	6-OH	6-OH	6-OH	5-OH	6-OH	6-OH	5-OH	6-OH	
	Ile126(O)	6-OH		6-OH				6-OH	5-OH	6-OH	
	Ala128(N)	6-OH		6-OH					5-OH	6-OH	
	Glu129(OE1)	N	N	N	N	6-OH	N			N	
	Glu129(OE2)							N	6-OH	N	
	Asn187(ND2)	5-OH	5,6-OH	5-OH	5-OH		5,6-OH		4-OH		
	Asn187(OD)							5,6-OH	4-OH	5,6-OH	
	Glu188(OE1)	4,5-OH	4,5-OH	4,5-OH	4,5-OH		4,5-OH	4,5-OH		4,5-OH	
	Glu188(OE2)			4-OH					4-OH		
	Glu268(OE1)			4-OH		4-OH			4-OH		
	Glu268(OE2)		6-OH		6-OH		6-OH				
	Tyr333(OH)				N		N				
**13**	**13 score**	**25.37**	**46.53**	**43.62**	**25.53**	**45.76**	**43.10**	**17.65**	**56.23**	**39.34**	
	Tyr83(OH)		5-OH		5,6-OH	5-OH		6-OH		6-OH	
	Ile126(O)										
	Ala128(N)		5-OH					6-OH			
	Glu129(OE1)	6-OH	6-OH	6-OH		6-OH	6-OH	4-OH		N	
	Glu129(OE2)				N			5-OH			
	Asn187(ND2)				5-OH				6-OH	5-OH	
	Asn187(OD)								6-OH	5-OH	
	Glu188(OE1)		4-OH		4-OH			N			
	Glu188(OE2)			4-OH	4-OH	4-OH			5-OH	4-OH	
	Glu268(OE1)	4,5-OH	4-OH	4,5-OH	4-OH	4,5-OH	4,5-OH		6-OH	5-OH	
	Glu268(OE2)		4-OH		5-OH	4,5-OH					
	Tyr333(OH)	6-OH		6-OH			6-OH				

^a^ The color codes in this table are used to indicate the number of runs that share the same top exit pose. See text below for further explanation.

## Supplementary Materials

The following are available online at https://www.mdpi.com/1424-8247/12/2/47/s1.
