# Supplementary material for: Chain-Branched Polyhydroxylated Octahydro-1H-Indoles as Potential Leads against Lysosomal Storage Diseases
Source: Pharmaceuticals (Basel). 2019 Mar 29;12(2):47. doi: 10.3390/ph12020047 (PMC6631223; doi:10.3390/ph12020047)

Supplementary Materials

Chain-branched Polyhydroxylated Octahydro-*1H*-indoles, as Potential Leads against Lysosomal Storage Diseases

Juan C. Estévez[1,2], Marcos A. González[1,2], M. Carmen Villaverde[2], Yuki Hirokami[3], Atsushi Kato[3], Fredy Sussman[1], David Reza[1,2], Ramón J. Estévez[1,2].

[1] Centro Singular de Investigación en Química Biolóxica e Materiais Moleculares, Universidade de Santiago de Compostela,15782 Santiago de Compostela, Spain; juancarlos.estevez@usc.es (J.C.E.); mar.gonzalez.castro@gmail.com (M.A.G.); fredy.sussman@usc.es (F.S.); david.reza@gmail.com (D.R.)

[2] Departamento de Química Orgánica, Universidade de Santiago de Compostela, 15782 Santiago de Compostela, Spain; mc.villaverde@usc.es (M.C.V)

[3] Department of Hospital Pharmacy, University of Toyama, Toyama 930-0194, Japan; phy.1.phy.t@gmail.com (Y.H.); kato@med.u-toyama.ac.jp (A.K.)

* Correspondence: ramon.estevez@usc.es; Tel: +34881815731

Table of contents

1. X-Ray Crystal structure for compound **5** SI2-SI3

2. X-Ray Crystal structure for compound **7** SI4-SI5

3. ^{1}H NMR and ^{13}C NMR spectra for compounds:

4b, 5, 6, 7, 8, 9, 10, 11, 12, 13 SI6-SI5

Figure S1. Single Crystal XRD- for compound **5**, with thermal ellipsoids drawn at the 50% probability level and **CCDC: 1885010**

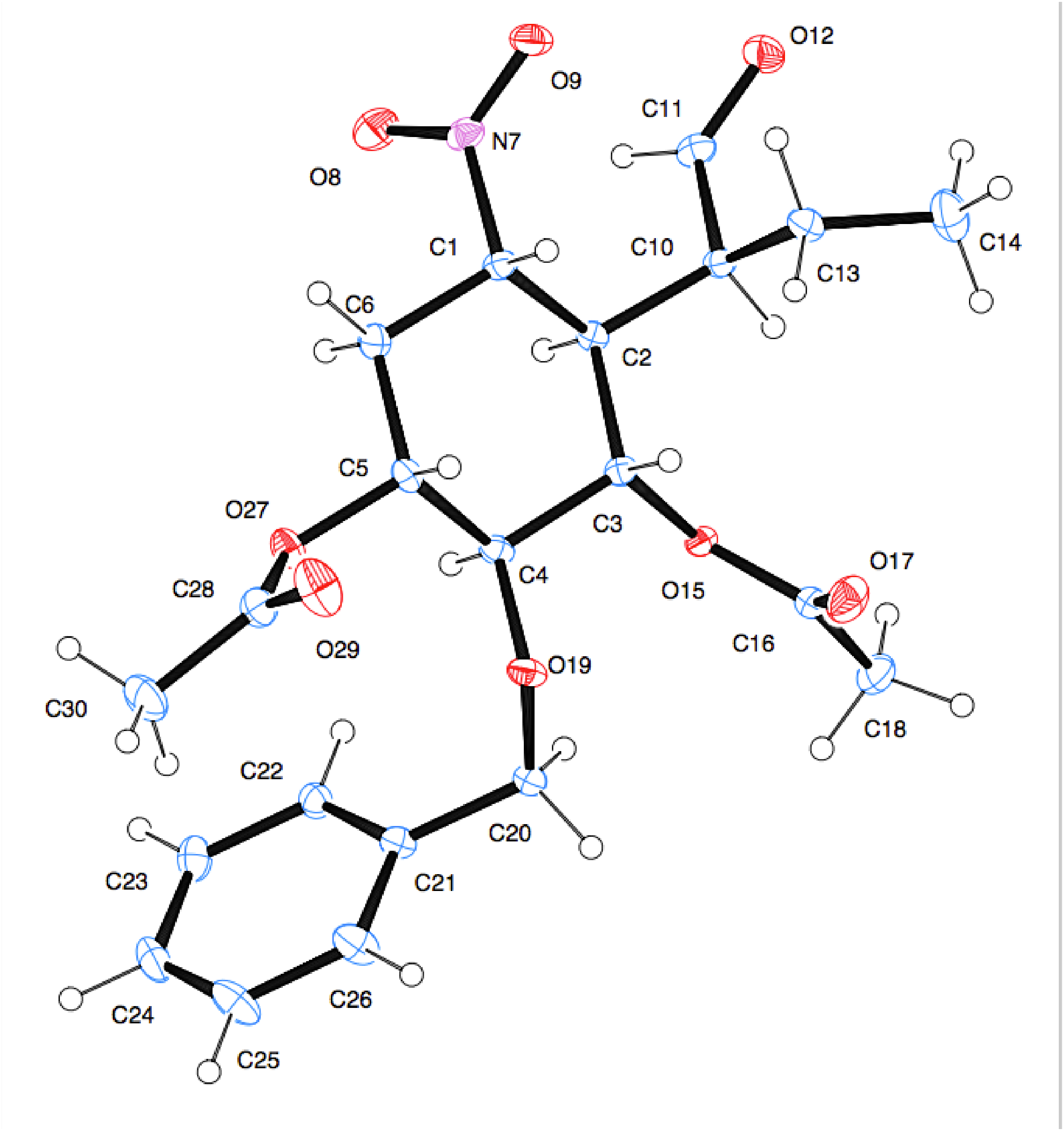

Table S1. Crystal data and structure refinement for compound **5**, and **CCDC: 1885010**

Experimental data **5** (2011re02mgc01)

**Crystal data**	
Chemical formula	$C_{21}H_{27}NO_8$
Temperature (K)	421.44
Crystal system, space group	Monoclinic *P*2$_1$
Temperature (K)	100(2) K
a,b,c(Å)	a = 10.4104(6) Å α= 90°<br>b = 8.8682(5) Å β= 101.541(3)°<br>c = 11.6487(6) Å γ= 90°
V(Å^3)	1053.68(10) Å^3
Z	2
Radiation type	*MoKa*
μ(mm^{-1})	0.102 mm^{-1}
Crystal size (mm)	0.42 x 0.14 x 0.09 mm^3
**Data collection**	
Diffractometer	BRUKER APPEX-II CCD
Absorption correction	Multi-scan<br>BRUKER *SADABS*
Tmin, *Tmax*	0.9253-0.9853
No. of measured, independent and observed (I > 2σ(I)] reflections	18089, 2777, 2302
**Rint**	0.052
(sin(θ)/λ)$_{max}$ {Å^{-1})	0.667
**Refinement**	
R[F^2 > 2σ(*F*^{2})], *wR(F^2)*, S	0.0390, 0.0878, 1.038
No. of reflections	2777
No. of parameters	274
H-atom treatment	H atoms treated by constrained rcfinement
Δr_{max}, Δ_{min} {e Å^{-3})	0.199, -0.193
Absolute structure	Absolute structure cannot be determined reliably.
Absolute structure parameter	N/A

Computer programs: APPEX2 (BRUKER AXS, 2005), *S/R97* (Giacovazzo *et ai.J*, 1999, *SHELXL97* (ShcJdrick, 1997), *ORTEP-3 for Windows* (Farrugia,1997), *WinGX* publication routines (Farrugia, 1999).

Figure S2. Single Crystal XRD- for compound **7**, with thermal ellipsoids drawn at the 50% probability level and **CCDC: 1885009**

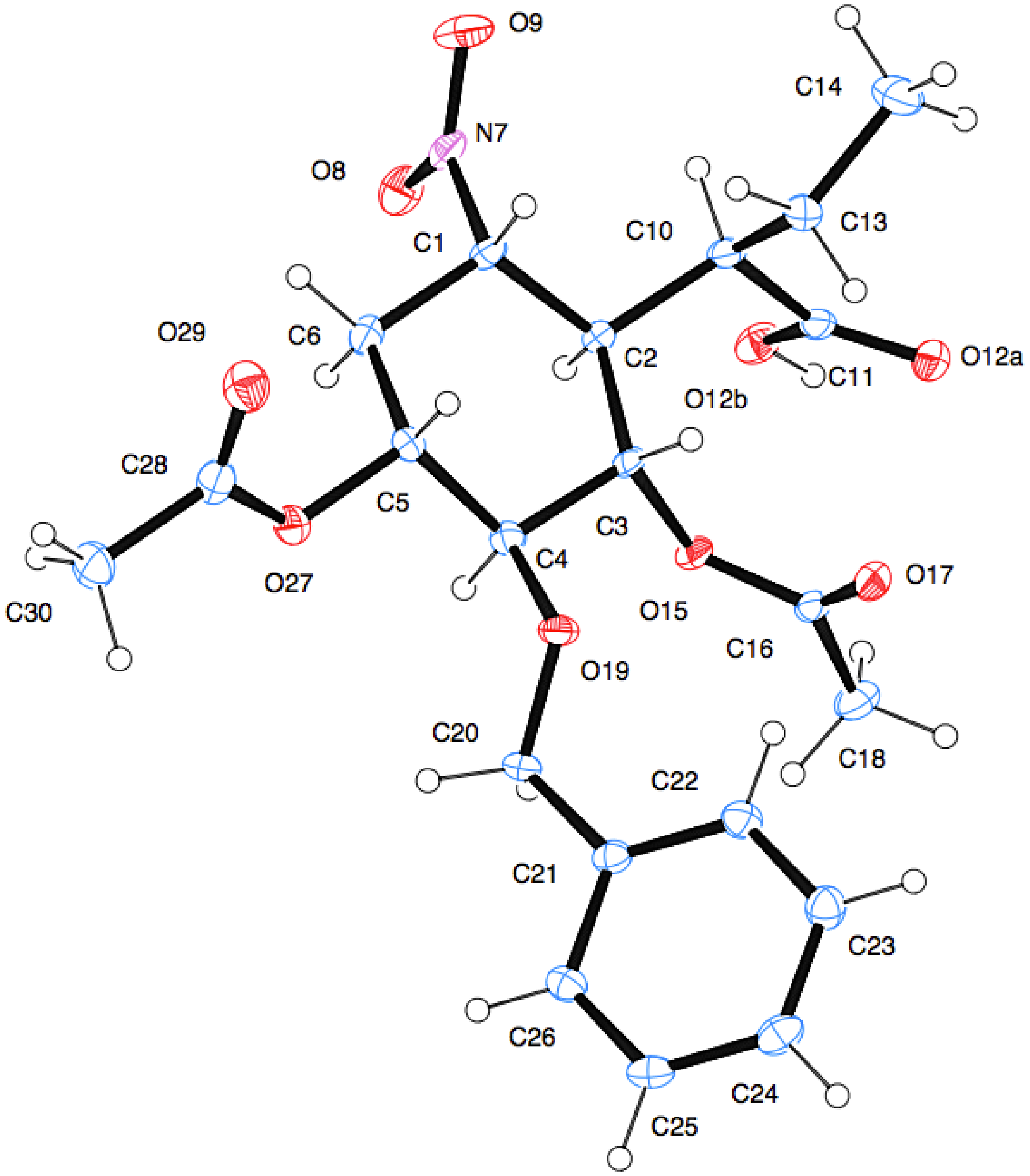

Table S2. Crystal data and structure refinement for compound **7**, and CCDC: **1885009**

Experimental data 7 (2011re03mgc02)

**Crystal data**	
Chemical formula	$C_{21}H_{27}NO_9$
Tcmperature (K)	437.44
Crystal system, space group	Monoclinic *P*2_1
Tcmpcrature (K)	100(2) K
a,b,c (Á)	a = 9.7916(13) Å α= 90°<br>b = 9.2932(12) Å β= 99.230(8)°<br>c = 12.3910(17) Å γ= 90°
V($Å^3$)	1112.9(3)$Å^3$
Z	2
Radiation type	*MoKa*
μ(mm^{-1})	0.103 mm^{-1}
Crystal size (mm)	0.35 x 0.12 x 0.04 mm^3
**Data collection**	
Diffractometer	BRUKER APPEX-II CCD
Absorption correction	Multi-scan<br>BRUKER *SADABS*
Tmin, Tmax	0.8784-0.9866
No. of measured, independent and observed [I > 2σ(I)] reflections	21663, 2417, 1945
**Rint**	0.0602
$(\sin(\theta)/\lambda)_{max}$ {$Å^{-1}$)	0.625
**Refinement**	
$R[F^2 > 2\sigma(F^2)]$, $wR(F^2)$, S	0.0384, 0.0913, 1.067
No. of reflections	2417
No. of parameters	287
H-atom treatment	H atoms treated by a mixture of indepcndent and constrained refinement
$\Delta□_{max}$, $\Delta□_{min}$ {e $Å^{-3}$)	0.171, -0.205
Absolute structure	Absolute structure cannot be determined reliably.
Absolute structure parameter	N/A

Computer programs: APPEX2 (BRUKER AXS, 2005), *S/R97* (Giacovazzo *et ai.J*, 1999, *SHELXL97* (ShcJdrick, 1997), *ORTEP-3 for Windows* (Farrugia, 1997), *WinGX* publication routines (Farrugia, 1999).

SI6. ^{1}H NMR (250 MHz, Cl_3CD, **4b**)

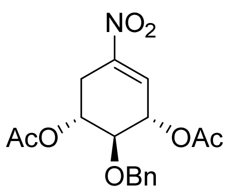

4b

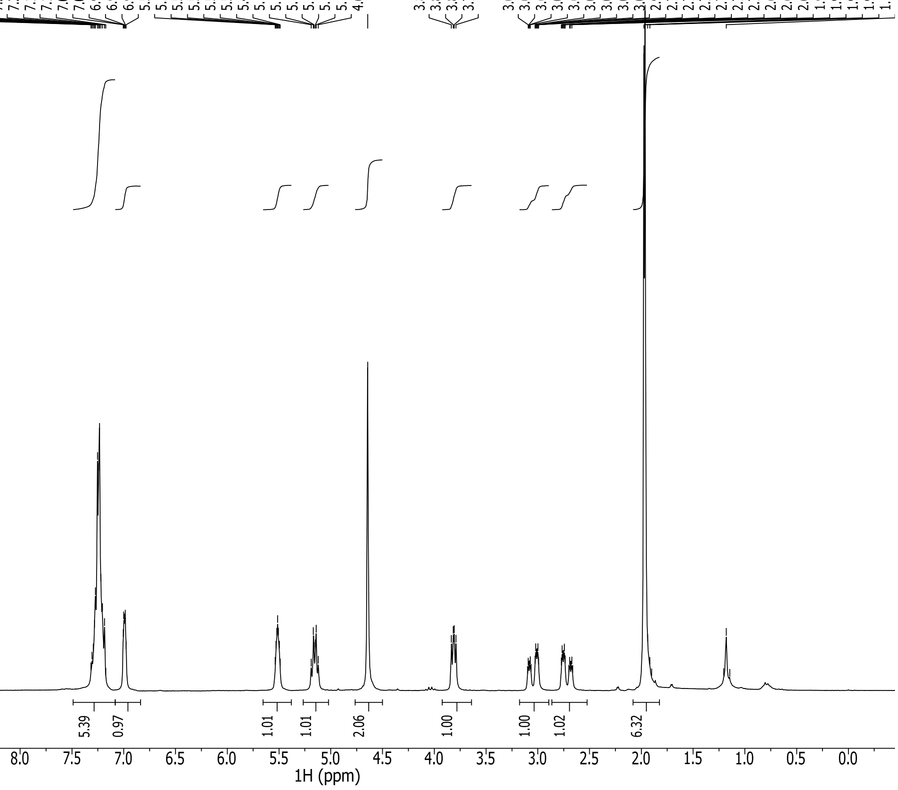

^{13}C NMR (62.5 MHz, Cl_3CD, **4b**)

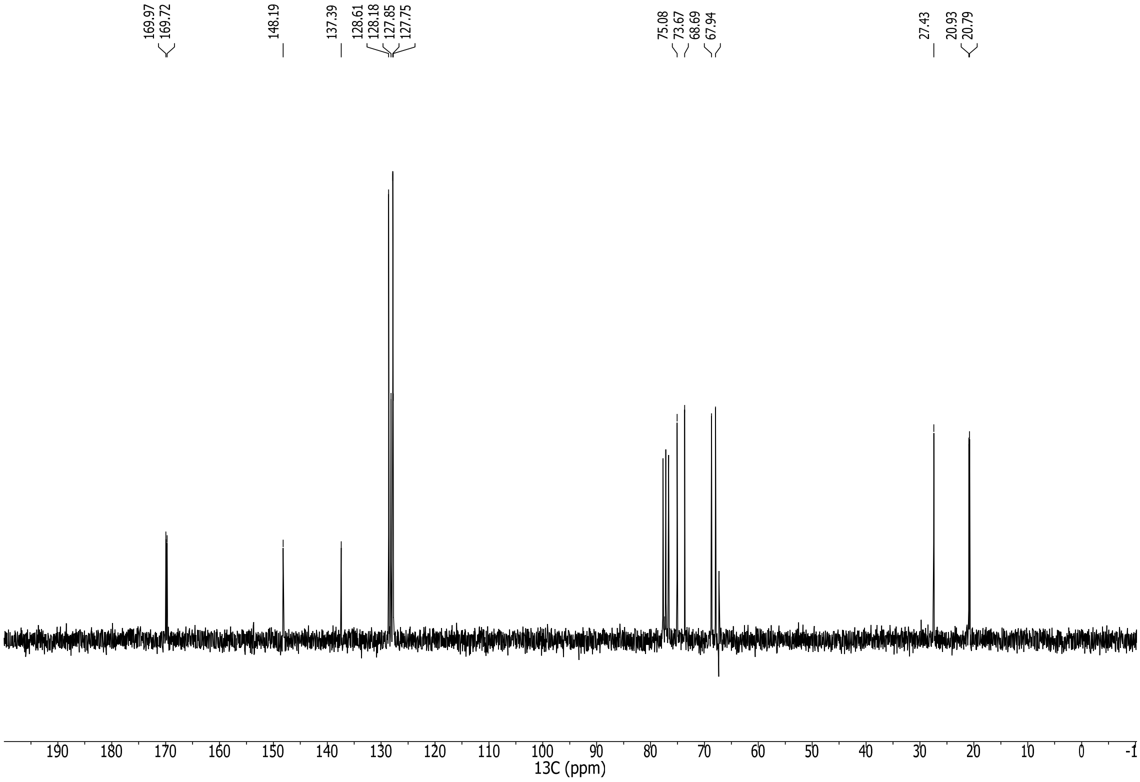

SI6. ^{1}H NMR (250 MHz, Cl_3CD, **5**)

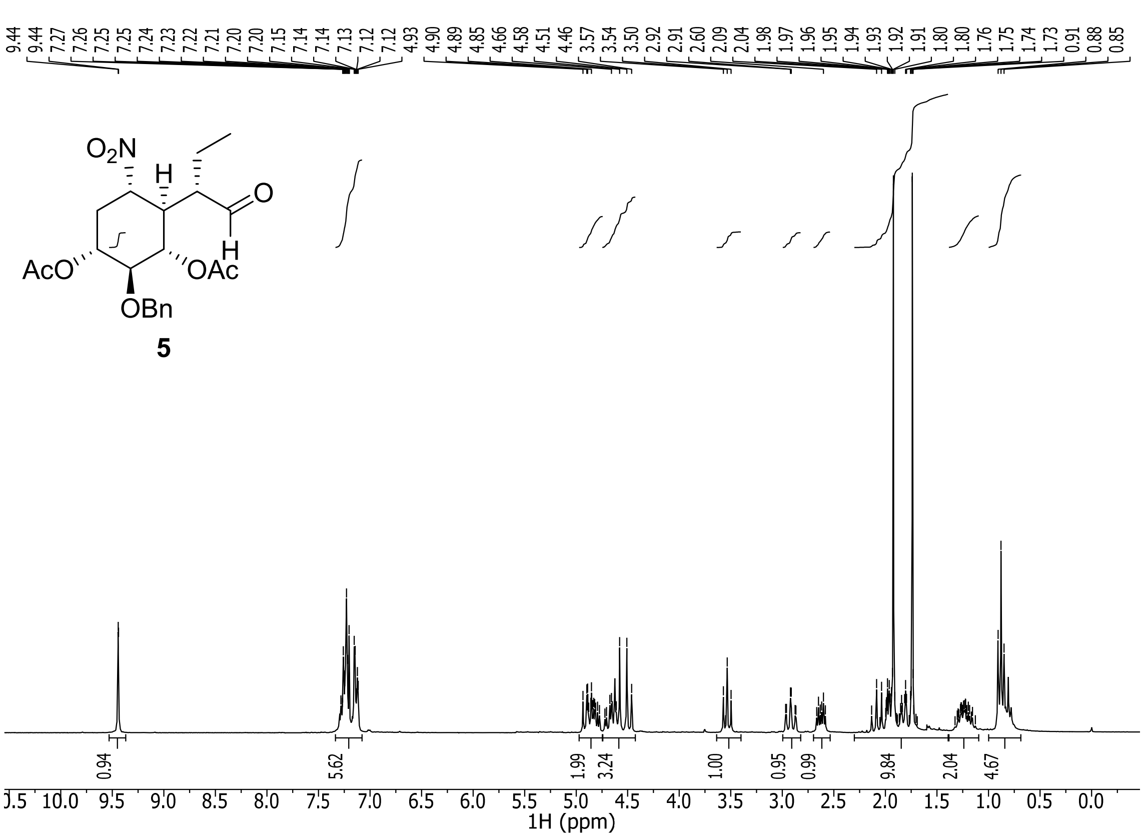

^{13}C NMR (62.5 MHz, Cl_3CD, **5**)

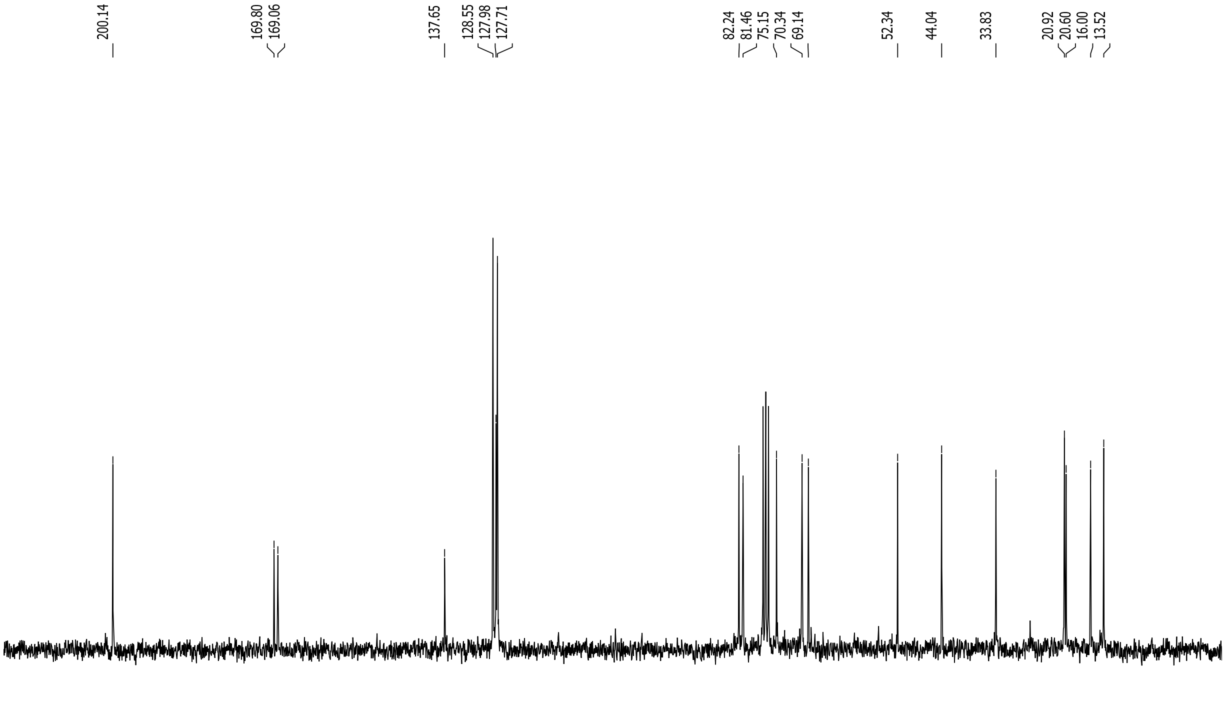

SI6. ^{1}H NMR (250 MHz, Cl_3CD, **6**)

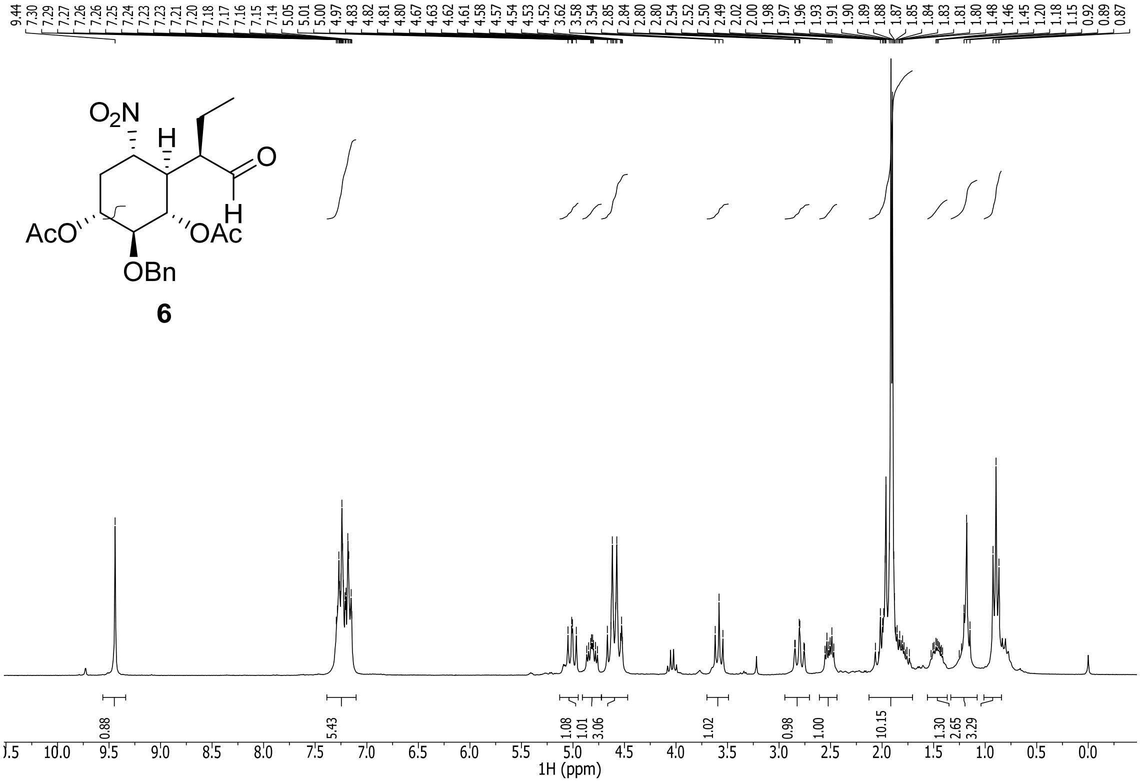

^{13}C NMR (62.5 MHz, Cl_3CD, **6**)

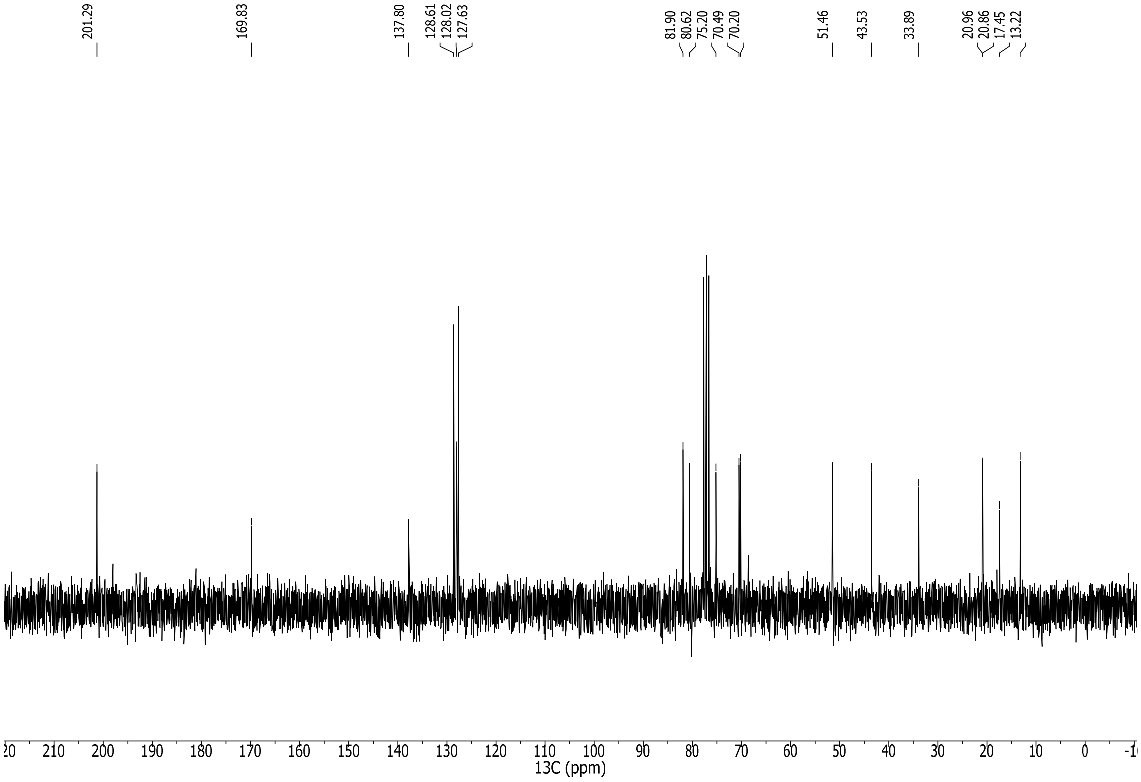

SI6. ^{1}H NMR (250 MHz, Cl3CD, **7**)

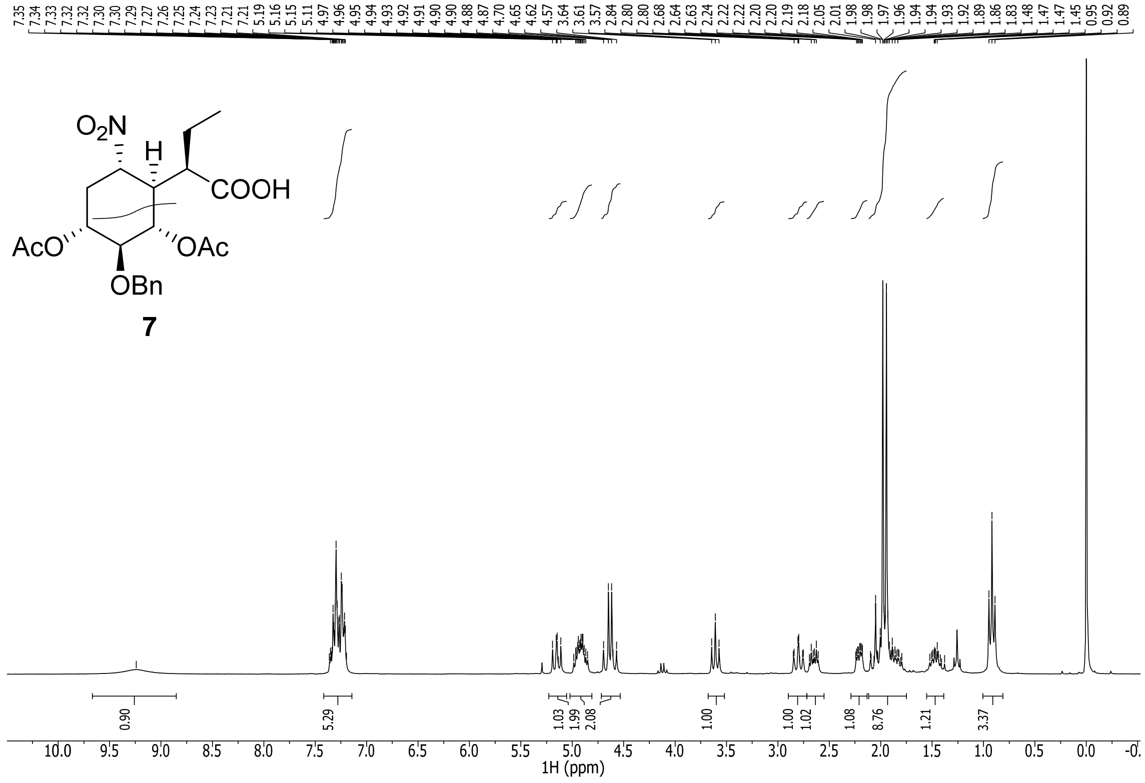

^{13}C NMR (62.5 MHz, Cl3CD, 7)

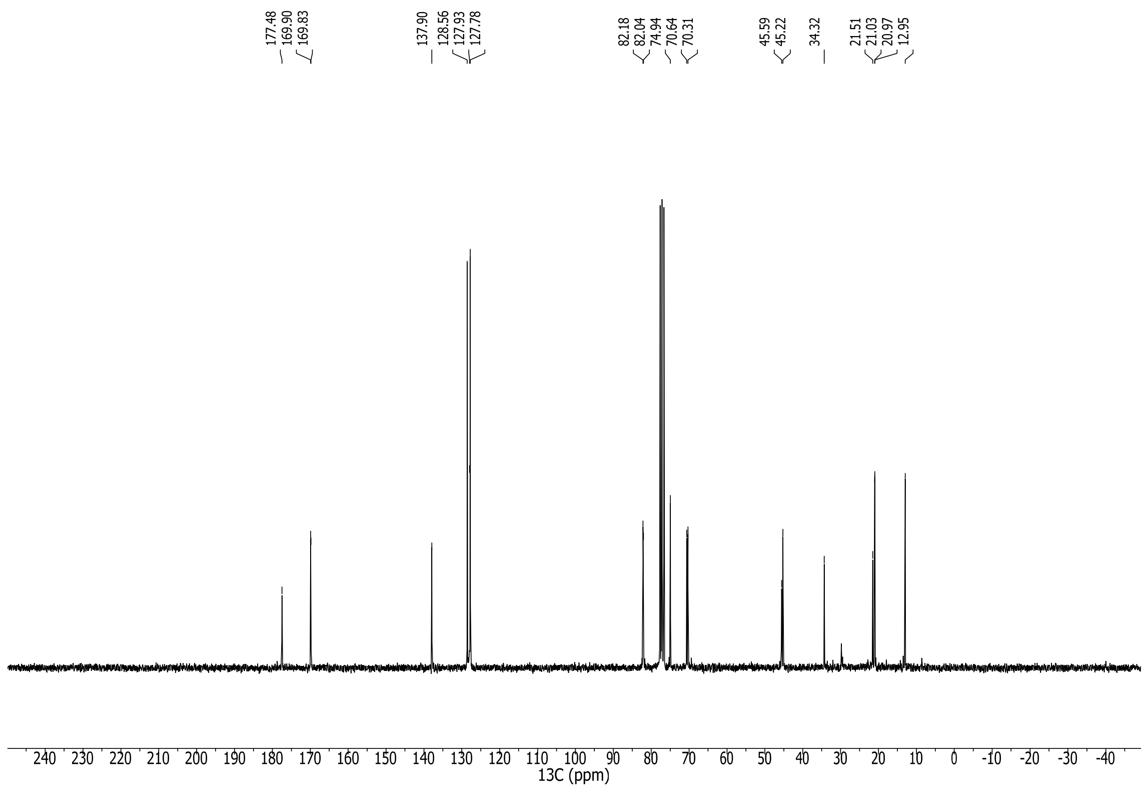

SI6. ^{1}H NMR (250 MHz, Cl_3CD, **8**)

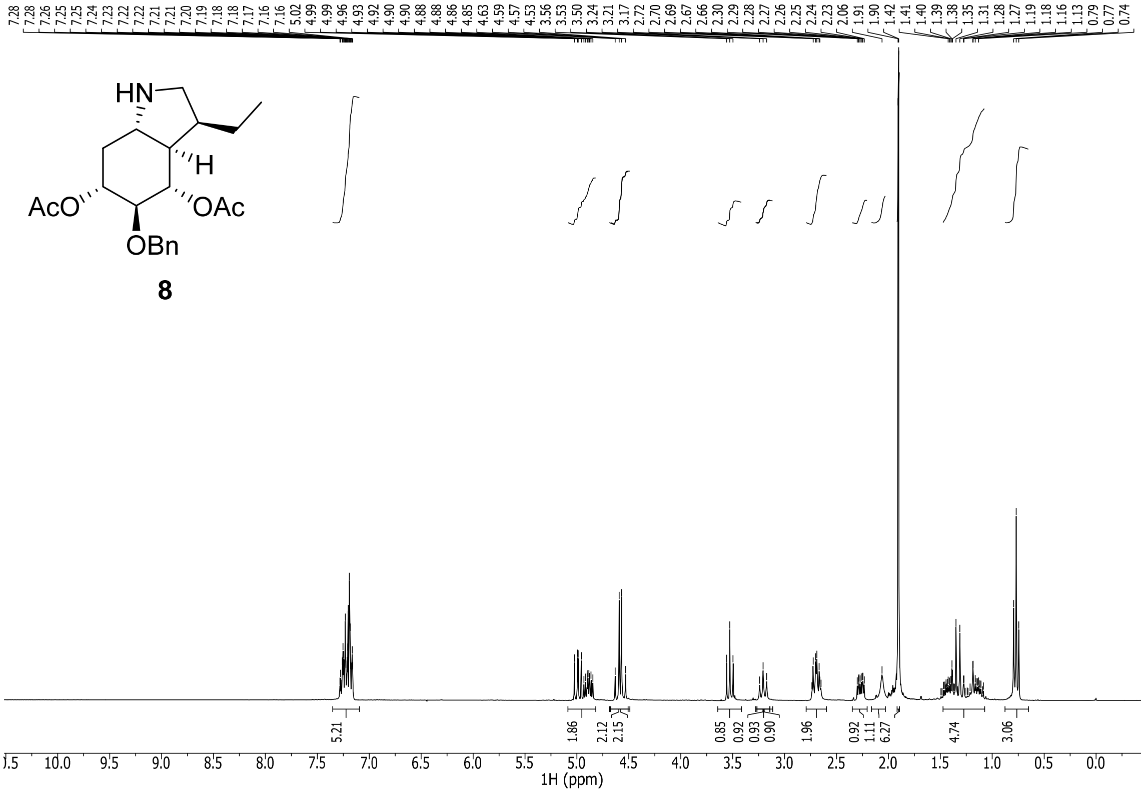

8

^{13}C NMR (62.5 MHz, Cl_3CD, **8**)

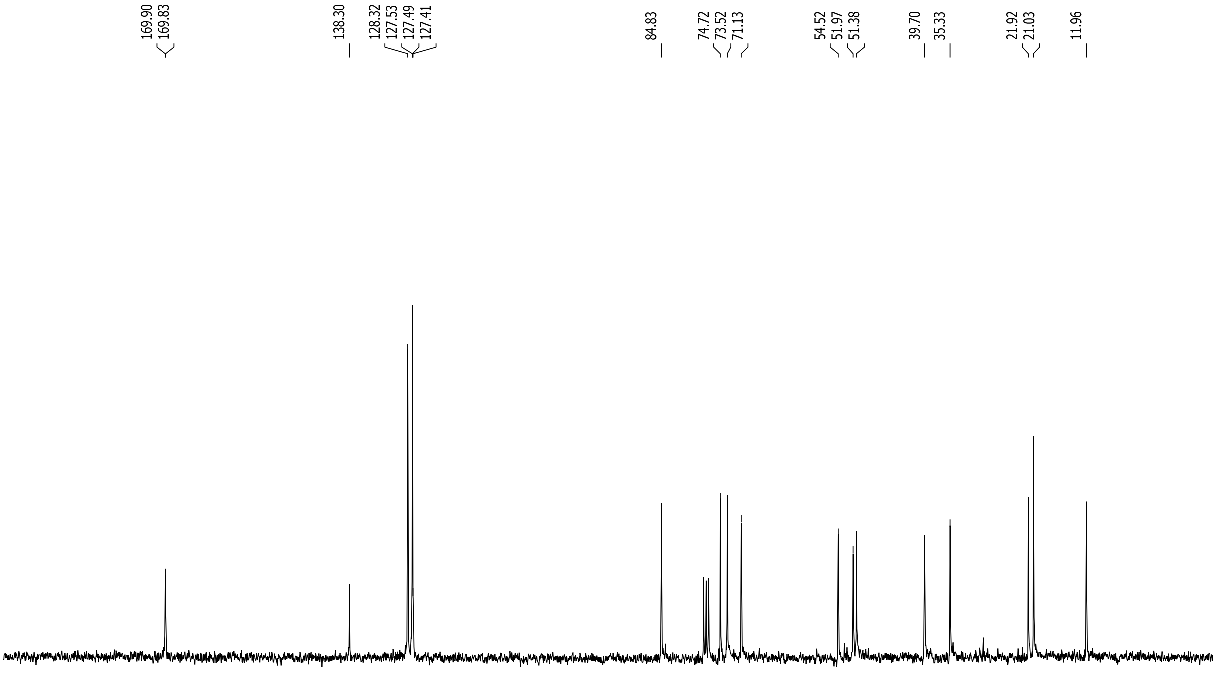

SI6. ¹H NMR (250 MHz, CD_3OD, **9**)

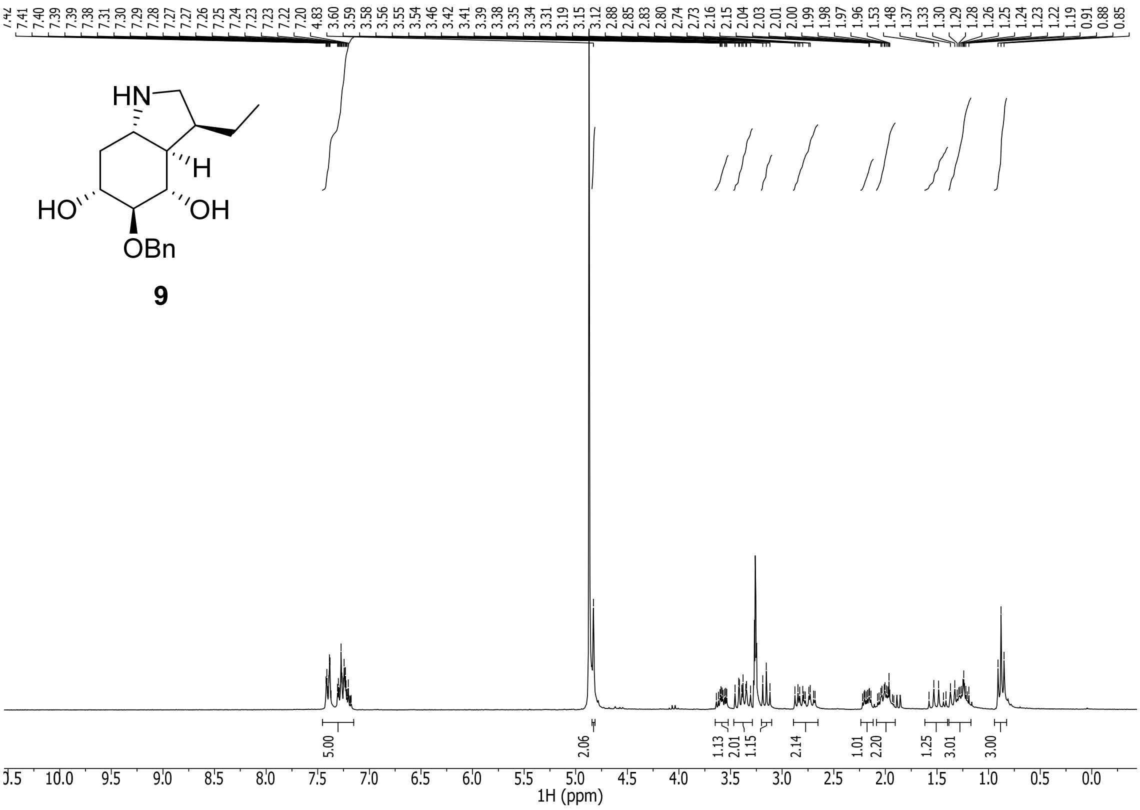

¹³C NMR (62.5 MHz, Cl_3CD, **9**)

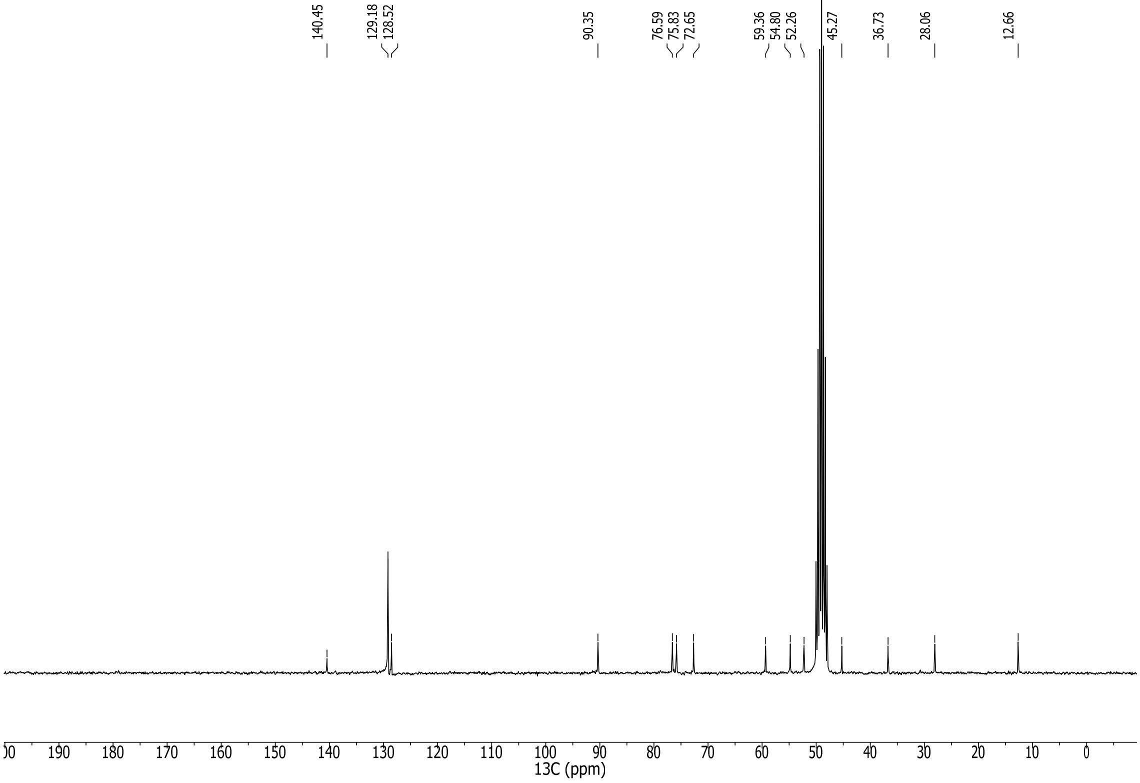

SI6. ^{1}H NMR (250 MHz, CD_3OD, **10**)

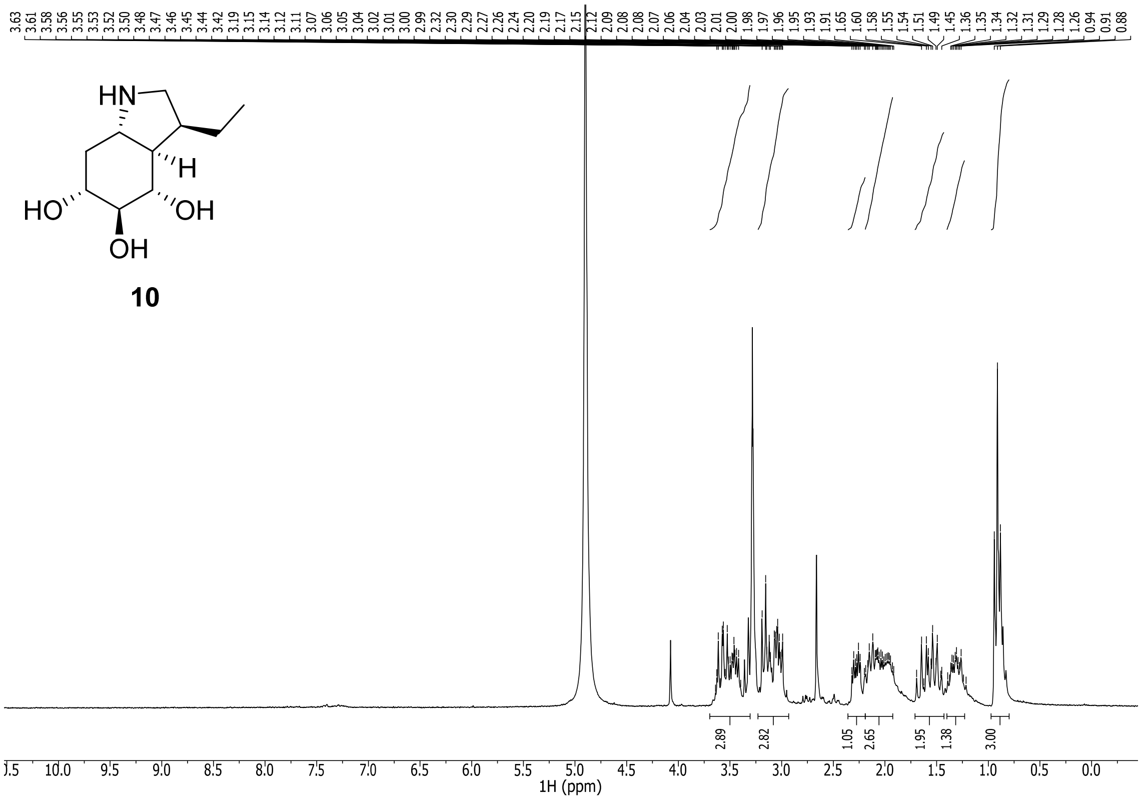

^{13}C NMR (62.5 MHz, Cl_3CD, **10**)

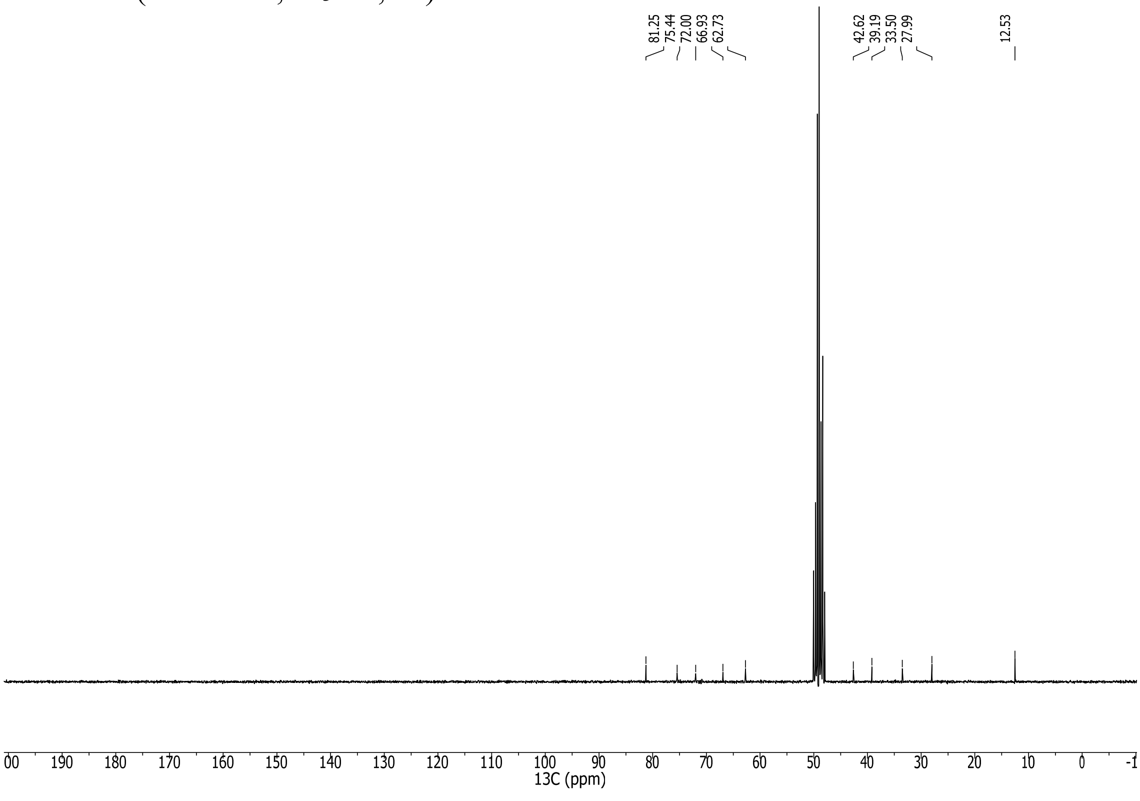

SI6. ^{1}H NMR (250 MHz, Cl_3CD, **11**)

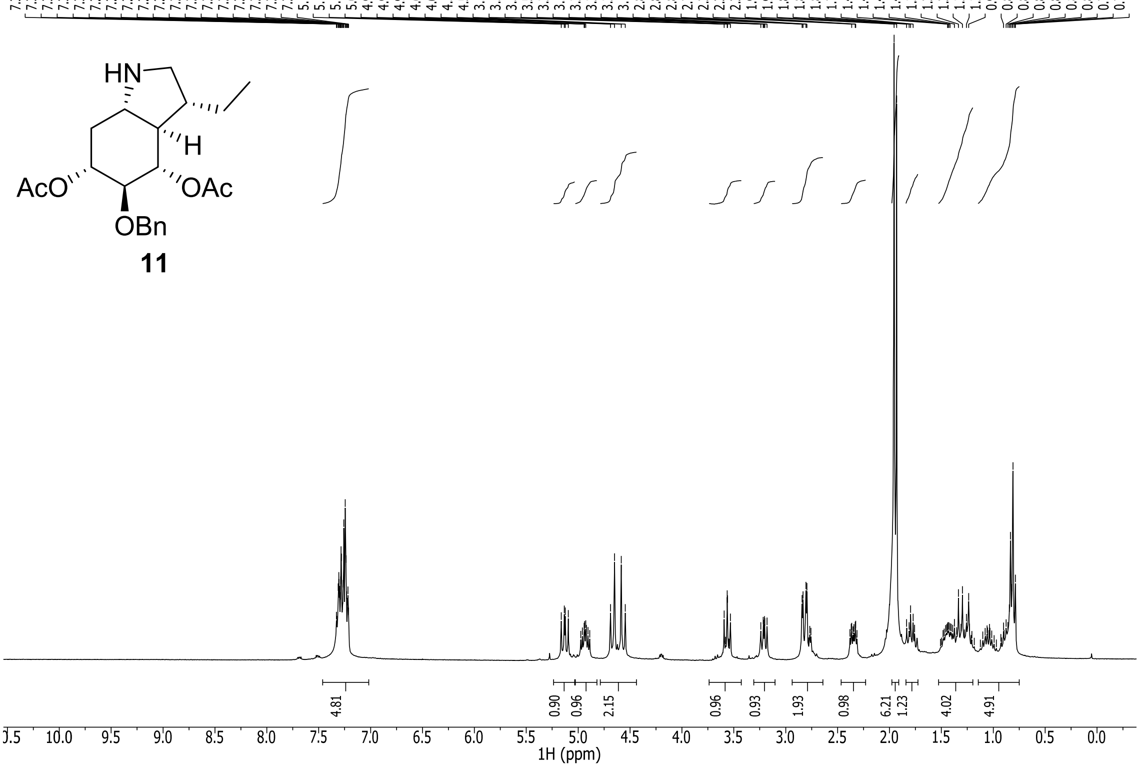

^{13}C NMR (62.5 MHz, Cl_3CD, **11**)

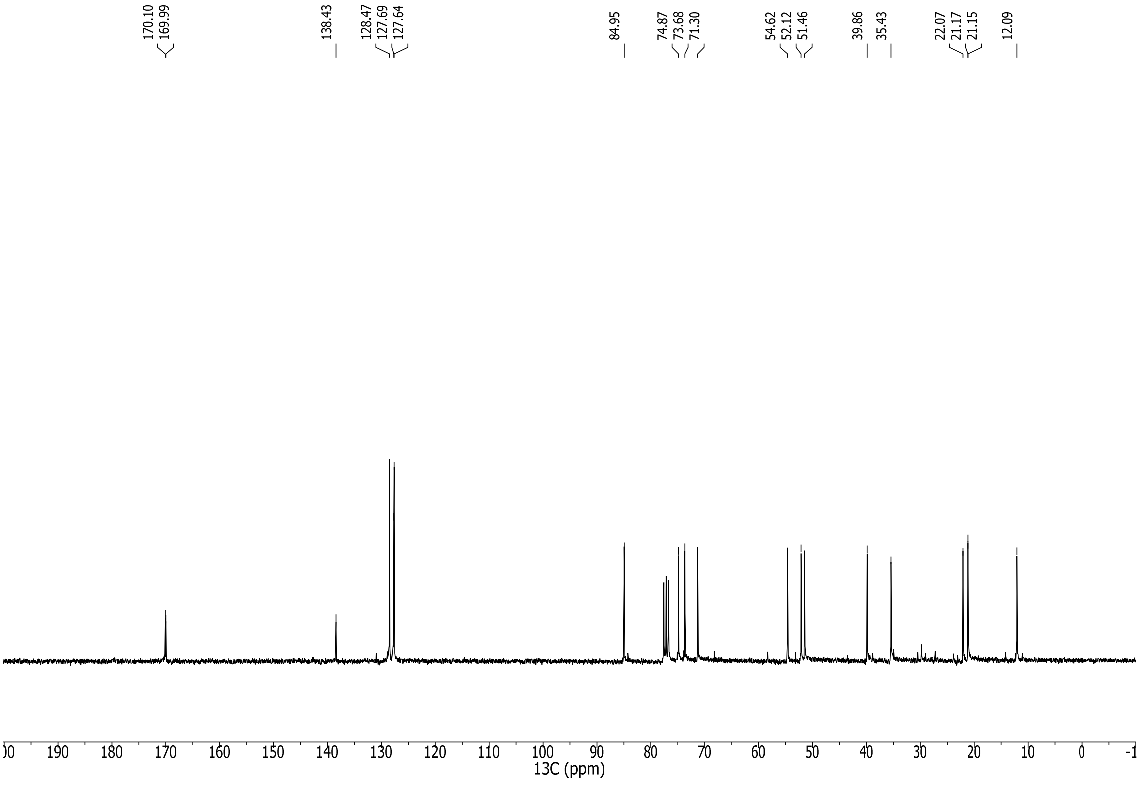

SI6. ^{1}H NMR (250 MHz, CD_3OD, **12**)

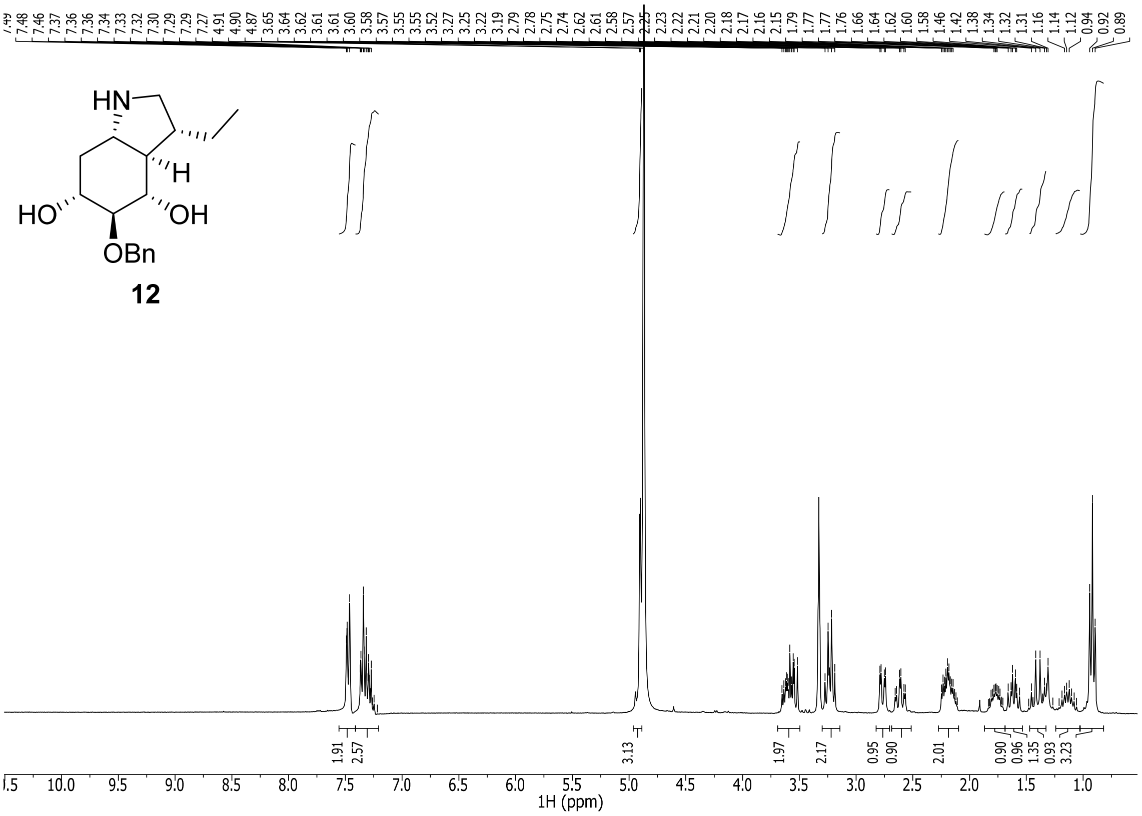

^{13}C NMR (62.5 MHz, Cl_3CD, **12**)

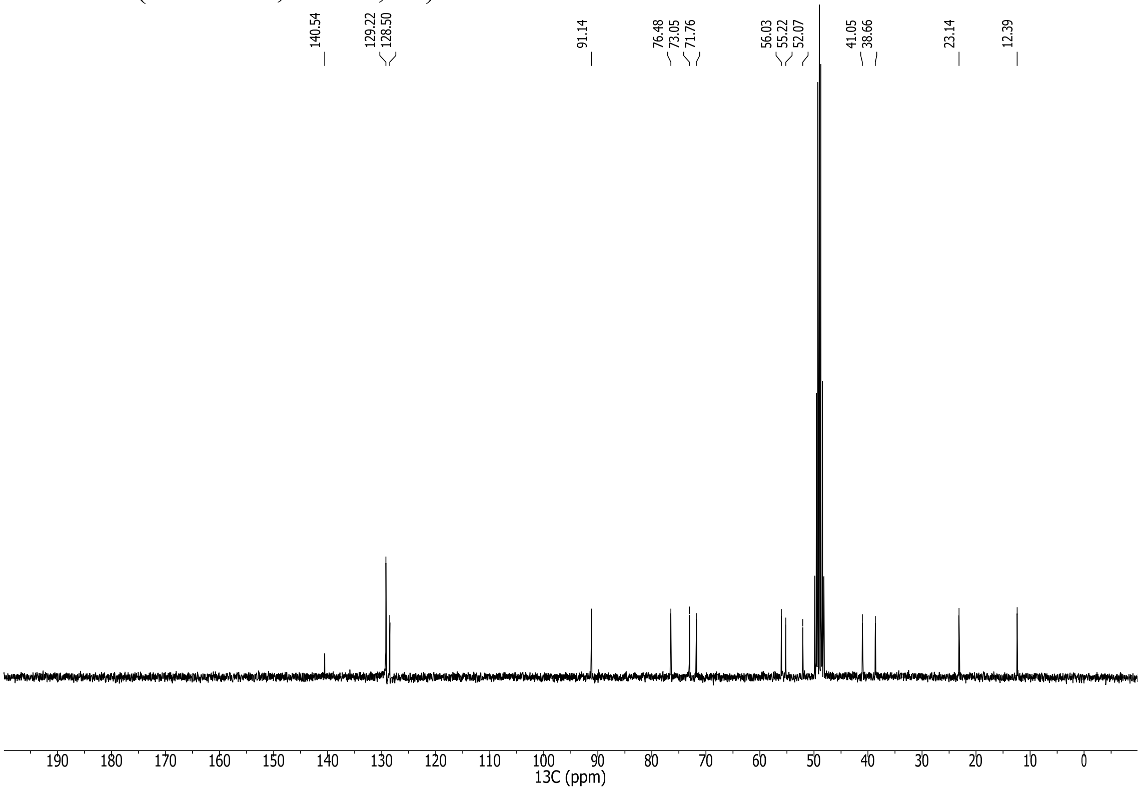

SI6. ^{1}H NMR (250 MHz, CD_3OD, **13**)

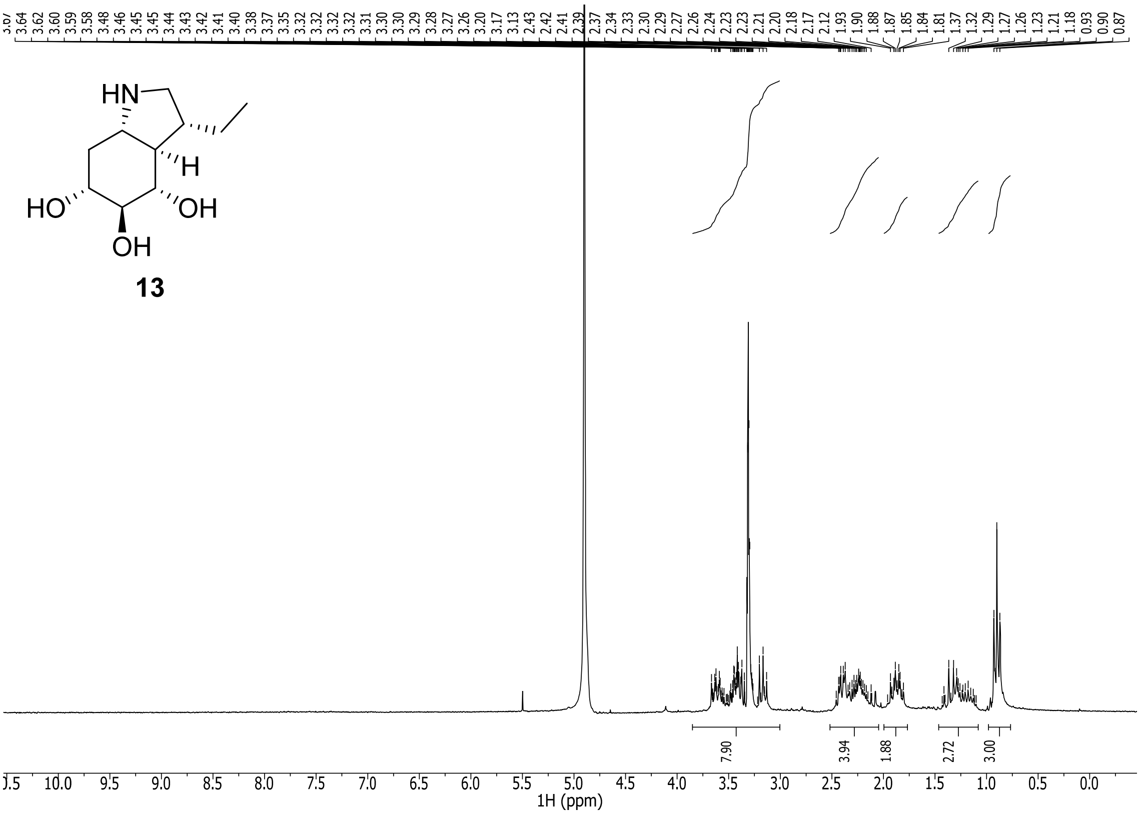

^{13}C NMR (62.5 MHz, Cl_3CD, **13**)

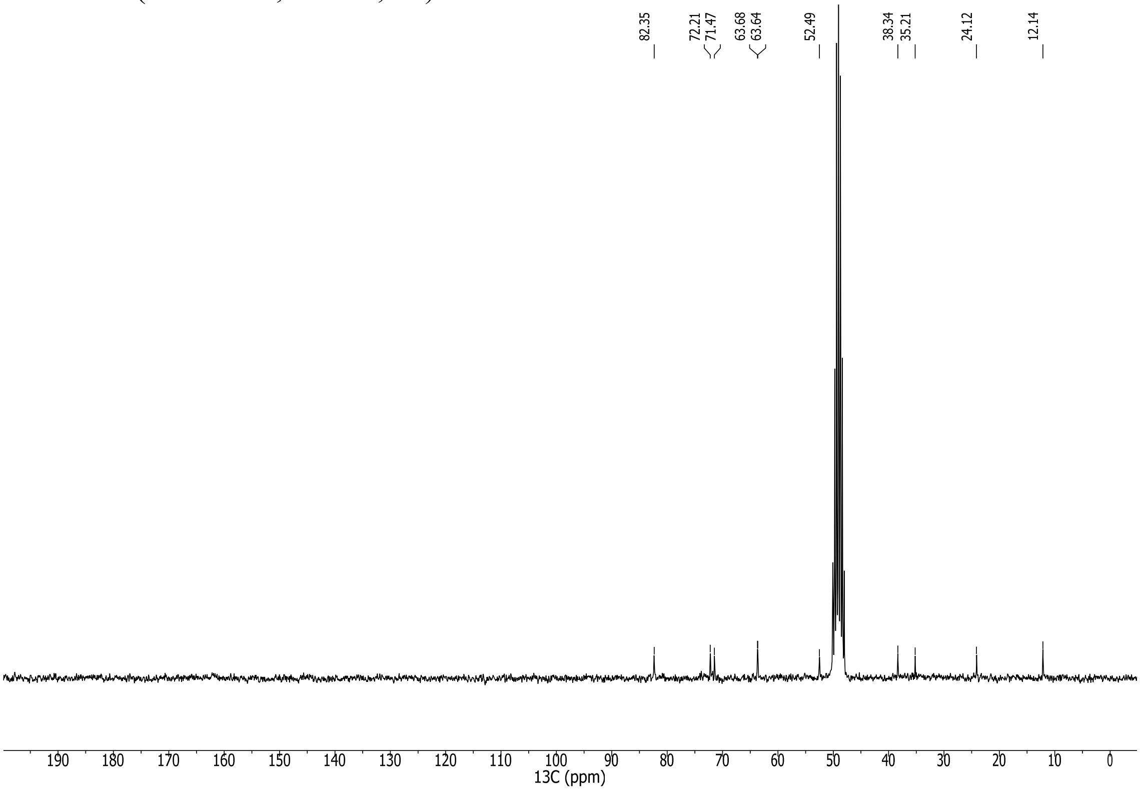

Supplement: Supplementary file 1 [file pharmaceuticals-12-00047-s001.pdf]
